# Super enhancers targeting ZBTB16 in osteogenesis protect against osteoporosis

**DOI:** 10.1038/s41413-023-00267-8

**Published:** 2023-06-07

**Authors:** Wenhui Yu, Zhongyu Xie, Jinteng Li, Jiajie Lin, Zepeng Su, Yunshu Che, Feng Ye, Zhaoqiang Zhang, Peitao Xu, Yipeng Zeng, Xiaojun Xu, Zhikun Li, Pei Feng, Rujia Mi, Yanfeng Wu, Huiyong Shen

**Affiliations:** 1 Department of Orthopedics, The Eighth Affiliated Hospital, Sun Yat-sen University, Shenzhen, 518003 PR China; 2 Shenzhen Key Laboratory of Ankylosing Spondylitis, Shenzhen, 518003 PR China; 3 Department of Orthopedics, Sun Yat-sen Memorial Hospital, Sun Yat-sen University, Guangzhou, 510120 PR China; 4 Center for Biotherapy, The Eighth Affiliated Hospital, Sun Yat-sen University, Shenzhen, 518003 PR China

**Keywords:** Osteoporosis, Bone, Pathogenesis

## Abstract

As the major cell precursors in osteogenesis, mesenchymal stem cells (MSCs) are indispensable for bone homeostasis and development. However, the primary mechanisms regulating osteogenic differentiation are controversial. Composed of multiple constituent enhancers, super enhancers (SEs) are powerful cis-regulatory elements that identify genes that ensure sequential differentiation. The present study demonstrated that SEs were indispensable for MSC osteogenesis and involved in osteoporosis development. Through integrated analysis, we identified the most common SE-targeted and osteoporosis-related osteogenic gene, *ZBTB16*. *ZBTB16*, positively regulated by SEs, promoted MSC osteogenesis but was expressed at lower levels in osteoporosis. Mechanistically, SEs recruited bromodomain containing 4 (BRD4) at the site of *ZBTB16*, which then bound to RNA polymerase II-associated protein 2 (RPAP2) that transported RNA polymerase II (POL II) into the nucleus. The subsequent synergistic regulation of POL II carboxyterminal domain (CTD) phosphorylation by BRD4 and RPAP2 initiated *ZBTB16* transcriptional elongation, which facilitated MSC osteogenesis via the key osteogenic transcription factor SP7. Bone-targeting ZBTB16 overexpression had a therapeutic effect on the decreased bone density and remodeling capacity of *Brd4*^fl/fl^ *Prx1*-cre mice and osteoporosis (OP) models. Therefore, our study shows that SEs orchestrate the osteogenesis of MSCs by targeting *ZBTB16* expression, which provides an attractive focus and therapeutic target for osteoporosis.

**Figure:**
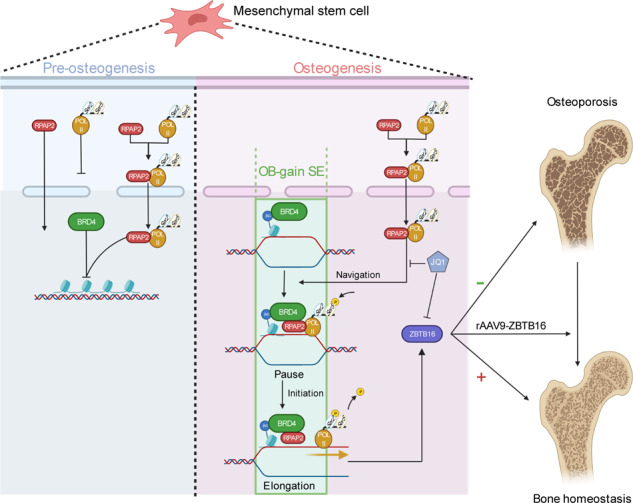
Without SEs located on osteogenic genes, BRD4 is not able to bind to osteogenic identity genes due to its closed structure before osteogenesis. During osteogenesis, histones on osteogenic identity genes are acetylated, and OB-gain SEs appear, enabling the binding of BRD4 to the osteogenic identity gene ZBTB16. RPAP2 transports RNA Pol II from the cytoplasm to the nucleus and guides Pol II to target ZBTB16 via recognition of the navigator BRD4 on SEs. After the binding of the RPAP2-Pol II complex to BRD4 on SEs, RPAP2 dephosphorylates Ser5 at the Pol II CTD to terminate the transcriptional pause, and BRD4 phosphorylates Ser2 at the Pol II CTD to initiate transcriptional elongation, which synergistically drives efficient transcription of ZBTB16, ensuring proper osteogenesis. Dysregulation of SE-mediated ZBTB16 expression leads to osteoporosis, and bone-targeting ZBTB16 overexpression is efficient in accelerating bone repair and treating osteoporosis.

Without SEs located on osteogenic genes, BRD4 is not able to bind to osteogenic identity genes due to its closed structure before osteogenesis. During osteogenesis, histones on osteogenic identity genes are acetylated, and OB-gain SEs appear, enabling the binding of BRD4 to the osteogenic identity gene ZBTB16. RPAP2 transports RNA Pol II from the cytoplasm to the nucleus and guides Pol II to target ZBTB16 via recognition of the navigator BRD4 on SEs. After the binding of the RPAP2-Pol II complex to BRD4 on SEs, RPAP2 dephosphorylates Ser5 at the Pol II CTD to terminate the transcriptional pause, and BRD4 phosphorylates Ser2 at the Pol II CTD to initiate transcriptional elongation, which synergistically drives efficient transcription of ZBTB16, ensuring proper osteogenesis. Dysregulation of SE-mediated ZBTB16 expression leads to osteoporosis, and bone-targeting ZBTB16 overexpression is efficient in accelerating bone repair and treating osteoporosis.

## Introduction

Mesenchymal stem cells (MSCs) show trilineage differentiation because these cells differentiate into osteocytes, chondrocytes and adipocytes. As primary sources of osteoblasts, MSCs show osteogenic functions that are closely associated with bone homeostasis and development.^1^ Disruption of MSC osteogenic differentiation leads to skeletal diseases such as osteoporosis (OP),^2^ which results in major financial and physical burdens on patients. Therefore, investigation of the MSC osteogenic mechanism and identification of novel therapeutic targets for OP are needed. Although many related studies have been performed, differences in cell lines, experimental conditions and downstream mechanisms have generally resulted in contradictory findings. To overcome these obstacles in the translation of novel findings to clinical applications, researchers must address the most common targets and upstream mechanisms mediating the key steps of MSC osteogenesis.

Super enhancers (SEs) are composed of high-density clusters of traditional enhancers (TEs) that act synergistically to recruit a high density of transcription factors (TFs) and cofactors for efficient transcription. Histone H3 lysine 27 acetylation (H3K27ac), histone H3 lysine 4 monomethylation (H3K4me1), bromodomain containing 4 (BRD4) and mediator complex subunit 1 (MED1) were utilized by the ROSE algorithm to identify SEs. First identified in embryonic stem cells, SEs mediate pluripotent state maintenance by promoting the expression of pluripotent identity genes, including *Oct4*, *Sox2*, *Nanog* and *Klf4*.^3^ During cell differentiation, SEs bind to cell type-specific TFs for the subsequent recruitment of cofactors, chromatin remodelers and POL II to initiate the gene expression network dedicated to lineage commitment. Subsequent studies have revealed that after adipogenic stimulation, SEs are redistributed to the adipogenesis-promoting genes *Pparg* and *Cebpa*.^4^ A recent study showed that the enhancer RNAs transcribed by SEs initiated the POL II-mediated transcription of myogenic identity genes.^5^ SEs may have an important role in osteogenesis, but the detailed mechanisms remain largely unknown.

ZBTB16 was first identified in acute promyelocytic leukemia as a transcriptional suppressor of the t(11;17) translocation.^6^ Many studies have shown that during different biological processes, such as proliferation,^7^ differentiation^8^ and apoptosis^9^, ZBTB16 exerts dual effects on transcription. The expression pattern of ZBTB16 is highly tissue- and lineage-specific. For example, ZBTB16 expression is upregulated only in certain stages of cell development and differentiation, such as spermatogenesis^10^ and embryonic limb bud patterning,^11^ which demonstrates the indispensable role of ZBTB16 in cell fate commitment. Several recent studies have consistently indicated that ZBTB16 expression is positively related to MSC osteogenesis.^12,13^ However, the upstream regulatory mechanism of ZBTB16 expression is unclear.

In this study, we demonstrated that SEs targeting *ZBTB16* promoted MSC osteogenesis through BRD4/RPAP2/POL II complexes. ZBTB16 expression was decreased in OP MSCs, and bone-targeting ZBTB16 overexpression exerted a therapeutic effect on the decreased bone density and remodeling capacity in *Brd4*^fl/fl^ *Prx1*-cre mice as well as OP mouse models. Our findings clarified the detailed mechanism of SEs targeting ZBTB16 in osteogenesis and provided a novel therapeutic target for OP.

## Results

### SE profile analysis and identification of critical osteoblastogenesis (OB)-gain SEs

**Fig. 1:**
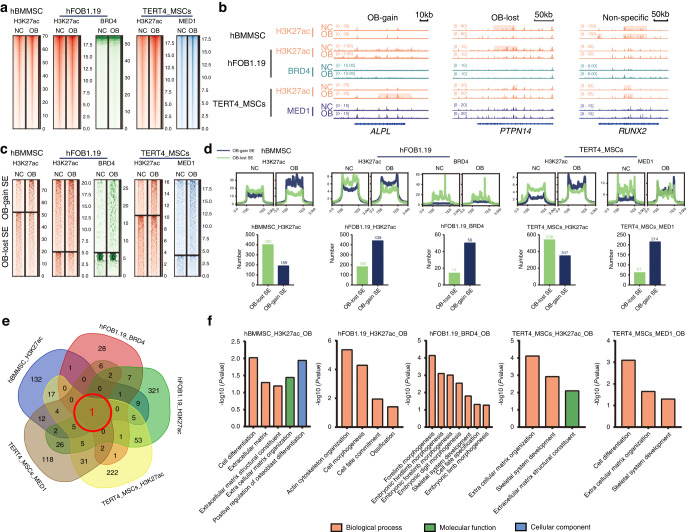
SE profile analysis and identification of critical OB-gain SEs. **a** ChIP-seq profile heatmaps showing H3K27ac abundance in hBMMSCs, H3K27ac and BRD4 abundance in hFOB1.19 cells and H3K27ac and MED1 abundance in immortal TERT4-MSCs. **b** Example signal traces of OB-gain, OB-lost and nonspecific SEs. The shadows indicate SE regions. **c** ChIP-seq profile heatmaps of the SEs identified by H3K27ac in hBMMSCs, H3K27ac and BRD4 in immortal hFOB1.19 and H3K27ac and MED1 in TERT4-MSCs cells. **d** The average SE signal levels are shown in line plots, and the numbers of OB-lost and OB-gain SEs are shown in histograms. **e** Venn diagram showing the intersecting OB-gain SEs from different datasets. **f** GO analyses of OB-gain SEs from different datasets

To avoid differences between cell lines with different experimental conditions and to explore the most universal SEs in osteogenesis, we used chromatin immunoprecipitation sequencing (ChIP-seq) data for the identification of different SE markers (including H3K27ac, BRD4 and MED1) in different osteogenic cells (including human bone marrow-derived MSCs, immortal TERT-MSCs and hFOB 1.19 cells). Our group generated ChIP-seq data for H3K27ac in MSCs, and other data were obtained from other studies, as noted in the Methods section.^14,15^ Heatmaps of enhancer ChIP-seq data signals showed the dynamics and distribution of enhancers between the normal control (NC) group without osteogenic induction and the osteoblastogenesis (OB) group with osteogenic induction (Fig. 1a). SEs were identified by the ROSE algorithm (Fig. S1), and SEs found only in the OB group were classified as OB-gain SEs (Fig. 1b). The profile heatmaps of SEs showed decreased enhancer signals in the OB-lost SEs and increased enhancer signals in the OB-gain SEs in the OB group (Fig. 1c). Notably, the SE profiles and differentially identified SEs were not consistent between different cell lines or different markers in one cell line (Fig. 1d and Table S1). A total of 189 OB-gain SEs and 400 OB-loss SEs were identified in the present study (Fig. 1d). To investigate the most universal SEs in five datasets, we performed Venn analysis, and only one common OB-gain SE, which was located in the *ZBTB16* locus, was found (Fig. 1e and Table S2). Gene Ontology (GO) functional analysis revealed that osteogenesis-related terms were enriched in the OB-gain SEs of different datasets, which indicated their importance in osteogenic differentiation (Fig. 1f).

### SEs are involved in MSC osteogenesis

**Fig. 2:**
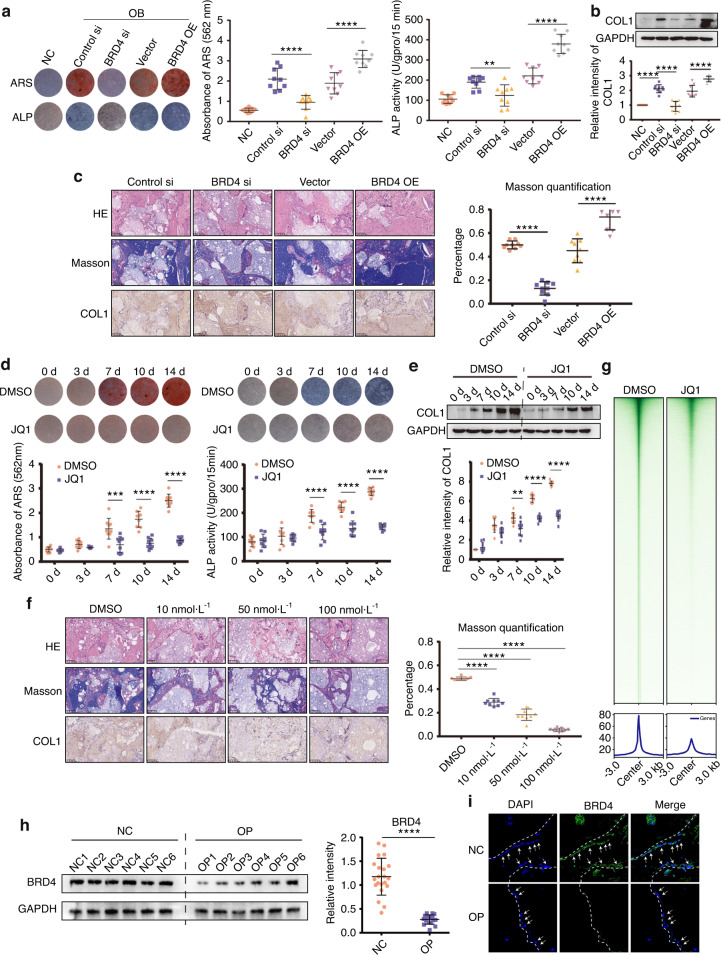
SEs are involved in MSC osteogenesis. **a** ARS and ALP staining showing that BRD4 knockdown and overexpression affect MSC osteogenesis. Quantification of ARS and ALP is shown in the scatter plots. **b** Western blot analysis showing that BRD4 knockdown and overexpression affect COL I expression in MSCs. **c** BRD4 knockdown and overexpression affect osteogenesis in vivo. HE, Masson and COL I immunohistochemistry staining of HA/TCP. Scatter plots showing Masson staining quantification. **d** ARS and ALP staining of MSCs treated with DMSO or 50 nmol·L^−1^ JQ1. Quantification of ARS and ALP is shown in the scatter plots. **e** COL I protein abundance in osteogenic-differentiating MSCs treated with DMSO or 50 nmol·L^−1^ JQ1. The relative intensity of COL I is shown in the scatter plot. **f** Effects of JQ1 on osteogenesis in vivo. HE, Masson and COL I immunohistochemistry staining of HA/TCP. Scatter plot showing Masson staining quantification. **g** CUT&Tag profile heatmap of BRD4 in MSCs treated with DMSO or 50 nmol·L^−1^ JQ1. **h** Western blot analysis showing BRD4 expression in MSCs from the NCs (*n* = 21) and OP patients (*n* = 17). **i** Immunofluorescence showing BRD4 expression in the femurs of the NCs (*n* = 21) and OP patients (*n* = 17). The statistical data are represented as the means ± SEMs, *n* = 9 (except **h**, **i**), *P < 0.05, **P < 0.01, ***P < 0.005, ****P < 0.001

BRD4 is one of the most important markers and effectors of SEs.^16^ To investigate the role of SEs in MSC osteogenesis, we constructed and verified BRD4 siRNAs and overexpression plasmids (Fig. S2A–C). Inhibiting BRD4 expression attenuated Alizarin Red S (ARS) and alkaline phosphatase (ALP) staining and their quantitative levels. Overexpression of BRD4 using plasmids increased the qualitative staining levels and quantitative levels (Fig. 2a). Western blot assays showed consistent results for collagen I (COL1) expression, which is necessary for bone formation, with the ARS and ALP assays (Fig. 2b). HE and Masson staining and less COL1 expression in the MSCs treated with the BRD4 siRNA compared to the MSCs treated with the control siRNA showed impaired osteogenesis. The opposite results were observed in the BRD4 OE group (Fig. 2c).

JQ1 is a BET inhibitor of BRD4 and has an inhibitory effect on SEs.^17^ Previous research revealed that JQ1 inhibited MSC proliferation,^18^ and we treated MSCs with JQ1 at a concentration gradient of 0–500 nmol·L^−1^ to verify the effect of JQ1 on proliferation. No significant difference was observed (Fig. S3A). ARS and ALP assays showed that MSCs treated with JQ1 at concentrations higher than 10 nmol·L^−1^ displayed significantly decreased osteogenic differentiation (Fig. S3B, C). To confirm the role of SEs in osteogenesis, we treated MSCs undergoing osteogenic differentiation with 50 nmol·L^−1^ JQ1 and measured the effects at different time points. JQ1 reduced the ARS and ALP staining intensities during osteogenic differentiation (Fig. 2d) and suppressed the expression of COL1 (Fig. 2e). JQ1 treatment at concentrations from 10 to 100 nmol·L^−1^ significantly inhibited the new bone formation of MSCs in the in vivo osteogenic assay (Fig. 2f). The general BRD4 cleavage under targets and tagmentation (CUT&Tag) signals shown by heatmaps and line plots were decreased in the JQ1 group, which indicated the inhibitory effect of JQ1 on the SEs of MSCs (Fig. 2g). BRD4 expression in MSCs from OP patients was significantly downregulated, as determined by both Western blot and immunofluorescence assays (Fig. 2h, i).

### SE disorder of MSCs leads to the OP phenotype and delayed bone repair

**Fig. 3:**
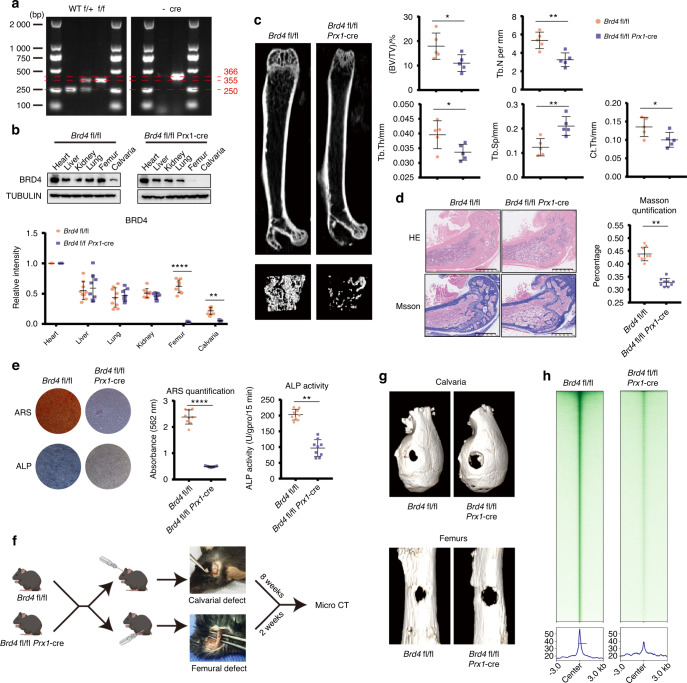
SE disorder of MSCs leads to the OP phenotype and delayed bone repair. **a** DNA electrophoresis was performed to genotype genetically modified mice. **b** Immunoblot analysis of BRD4 protein expression in different organs of the *Brd4*^fl/fl^ and *Brd4*^fl/fl^ *Prx1*-cre mice. Scatter plot showing the relative protein abundance of BRD4. **c** Micro-CT analysis of the *Brd4*^fl/fl^ and *Brd4*^fl/fl^ *Prx1*-cre mice, and the trabecular bones were 3D reconstructed. Bone morphometric analysis was performed, and the parameters included bone BV/TV, Tb. Th, Tb. N, Tb. Sp and cortical Ct. Th. **d** HE and Masson staining of femurs from the *Brd4*^fl/fl^ and *Brd4*^fl/fl^ *Prx1*-cre mice. Scatter plot showing the quantification of Masson staining. **e** ARS and ALP staining of osteogenic differentiating MSCs extracted from the *Brd4*^fl/fl^ and *Brd4*^fl/fl^ *Prx1*-cre mice. Quantification of ARS and ALP are shown in the scatter plots. **f** Diagram showing the workflow of calvarial and femoral defect induction and analysis. **g** Micro-CT analysis showing the calvarial and femoral defects of the *Brd4*^fl/fl^ and *Brd4*^fl/fl^ *Prx1*-cre mice. **h** CUT&Tag profile heatmap of BRD4 in MSCs from the *Brd4*^fl/fl^ and *Brd4*^fl/fl^ *Prx1*-cre mice. The statistical data are represented as the means ± SEMs, *n* = 9 (*n* = 5 in **c**), *P < 0.05, **P < 0.01, ***P < 0.005, ****P < 0.001

To verify the regulatory effect of SEs on MSC osteogenesis, we generated *Brd4*^fl/fl^ mice with the CRISPR‒Cas9 technique. We crossed *Brd4*^fl/fl^ mice with *Prx1*-cre mice to generate *Brd4*^fl/fl^ Prx1-cre mice with BRD4 conditional knockout (CKO) in MSCs (Fig. S4A). Genotyping (Fig. 3a) and detection of BRD4 expression in different tissues (Fig. 3b) verified the specific knockout of BRD4 in the skeletal system. Upon BRD4 knockout in MSCs, the *Brd4*^fl/fl^ *Prx1*-cre mice exhibited reduced trabecular bone size and thinner cortical bones than the *Brd4*^fl/fl^ mice, as shown by microcomputed tomography (micro-CT) analyses. Decreased bone volume/total volume (BV/TV), trabecular thickness (Tb. Th), trabecular number (Tb. N) and cortical bone thickness (Ct. Th) and increased trabecular spacing (Tb. Sp) were observed in the femurs of the *Brd4*^fl/fl^ *Prx1*-cre mice, indicating the disease phenotype of OP (Fig. 3c). HE and Masson staining also confirmed the OP phenotype of the *Brd4*^fl/fl^ *Prx1*-cre mice (Fig. 3d). MSCs from the *Brd4*^fl/fl^ *Prx1*-cre mice were extracted, and their weaker osteogenic differentiation was confirmed using ARS and ALP staining (Fig. 3e). Eight-week-old *Brd4*^fl/fl^ *Prx1*-cre mice and *Brd4*^fl/fl^ mice were used to create defects in the calvaria and femur. Eight weeks after generation of the calvarial defects and two weeks after generation of the femoral defects, the mice were sacrificed for micro-CT analysis (Fig. 3f). The *Brd4*^fl/fl^ *Prx1*-cre mice showed weaker bone repair for calvarial and femoral defects than the control mice (Fig. 3g and Fig. S4B). The general BRD4 CUT&Tag signals of MSCs isolated from the *Brd4*^fl/fl^ *Prx1*-cre mice were significantly lower, indicating a disordered SE signal in the MSCs from the BRD4 CKO mice (Fig. 3h). These results demonstrated that SE disorder in MSCs led to the OP phenotype and delayed bone repair.

### *ZBTB16* plays a pivotal role in SE-mediated osteogenesis but is decreased in OP

**Fig. 4:**
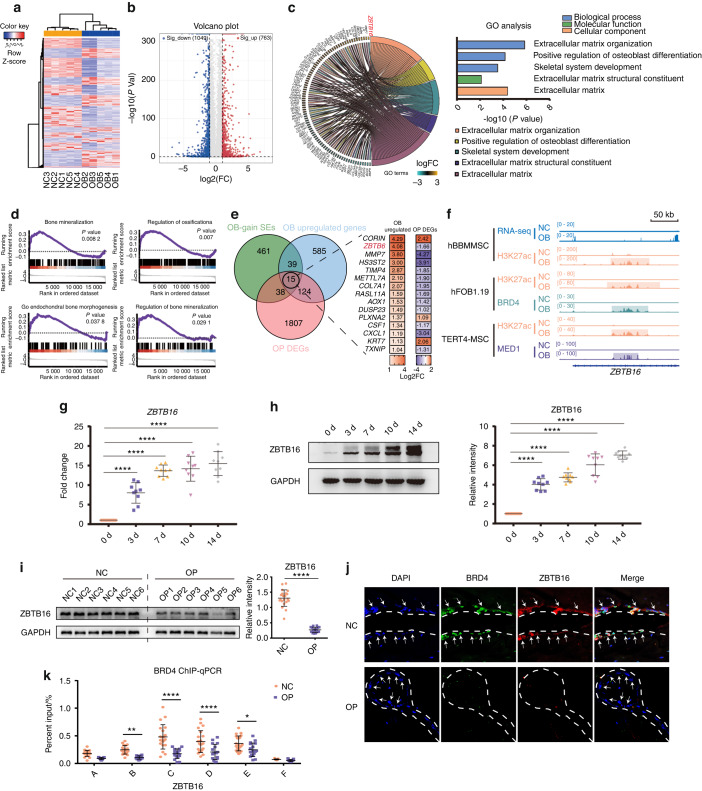
*ZBTB16* plays a pivotal role in SE-mediated osteogenesis but is decreased in OP. **a** Heatmap of DEGs between MSCs not undergoing osteogenic induction and MSCs during osteogenic differentiation. **b** Volcano plot showing the DEGs of MSCs in the OB and NC groups. **c** GO analysis showing the osteogenic-related terms. **d** GSEA showing the enriched osteogenic-related terms between the NC and OB groups. **e** Venn diagram showing the intersection of OB-gain SEs in all datasets, significantly upregulated genes in OB MSCs, and DEGs in OP MSCs compared to those of the NC subjects. The log2fc of the 15 intersected genes are shown. **f** Signal traces of RNA-seq and ChIP-seq data. The shadows show the SE regions. **g** Scatter plot showing the expression of ZBTB16 mRNA in osteogenic differentiating MSCs. **h** Immunoblot analysis showing the protein abundance of ZBTB16 in osteogenic differentiating MSCs. Scatter plot showing the relative abundance of ZBTB16. **i** Western blot analysis showing ZBTB16 expression in MSCs from the NCs (*n* = 21) and OP patients (*n* = 17). **j** Immunofluorescence showing BRD4 and ZBTB16 expression in the femurs of the NCs (*n* = 21) and OP (*n* = 17) patients. **k** ChIP‒qPCR analysis showing BRD4 occupancy on *ZBTB16* in MSCs of the NCs (*n* = 21) and OP (*n* = 17) patients. The statistical data are represented as the means ± SEMs, *n* = 9 (except **i**–**k**), *P < 0.05, **P < 0.01, ***P < 0.005, ****P < 0.001

To confirm the regulatory effect of SEs and identify the pivotal genes in this process, we performed RNA-seq of MSCs before and after osteogenic induction. The heatmap showed distinct expression profiles between the NC group without osteogenic induction and the OB group with osteogenic induction (Fig. 4a). We identified 1812 differentially expressed genes between the OB group and the NC group, among which 763 genes were upregulated and 1049 genes were downregulated (Fig. 4b & Table S3). Several osteogenesis-related term categories, including extracellular matrix organization, positive regulation of osteoblast differentiation, skeletal system development, extracellular matrix structural constituent and extracellular matrix, were enriched in the GO analysis (Fig. 4c). GO osteogenic terms and the log2fc values of the related genes are shown in a circle plot (Fig. 4c). Gene set enrichment analysis (GSEA) showed obvious enrichment of gene sets associated with osteogenesis, including bone mineralization, regulation of ossification, endochondral bone morphogenesis and regulation of bone mineralization (Fig. 4d). OB-gain SE-related genes, the significantly upregulated genes of OB MSCs from our RNA sequencing data and the differentially expressed genes of OP MSCs from Geng’s research^19^ were intersected, among which 15 intersected genes were identified (Fig. 4e and Table S4), including the common OB-gain SEs targeting *ZBTB16* (Fig. 1e). The ChIP-seq signal traces of *ZBTB16* are shown in Fig. 4f. ZBTB16 expression was confirmed to be upregulated upon osteogenic differentiation initiation (Fig. 4g, h). ZBTB16 expression in MSCs of OP patients was significantly downregulated (Fig. 4i), and the immunofluorescence results of bone tissue from OP patients also showed lower ZBTB16 expression in MSCs (Fig. 4j).

### ZBTB16 promotion of osteogenesis is regulated by BRD4 binding with RPAP2

**Fig. 5:**
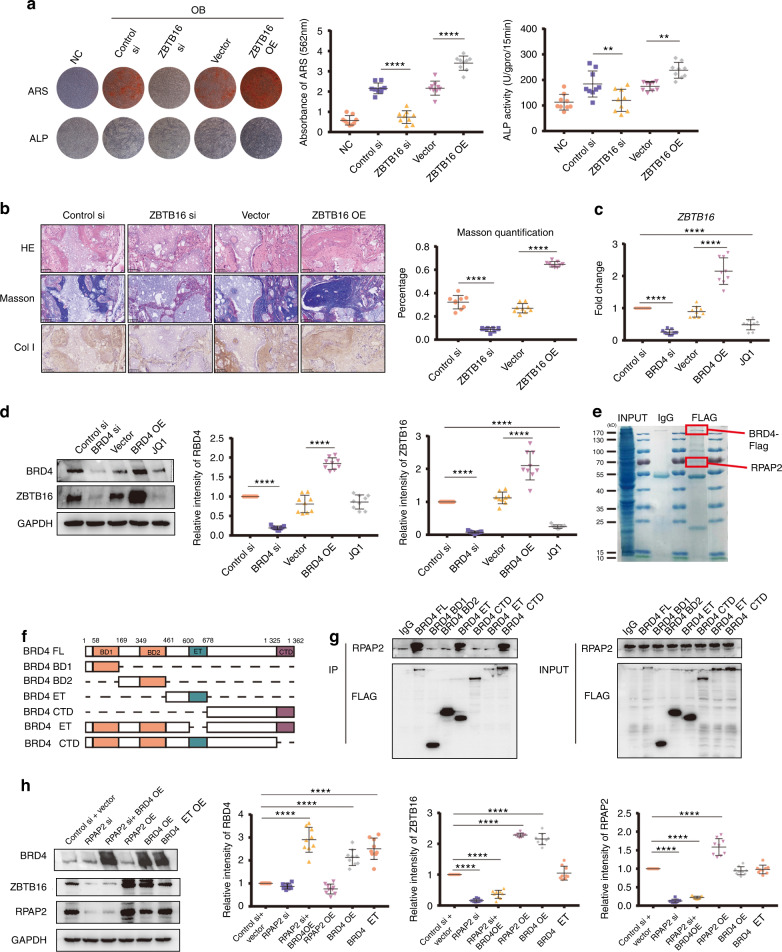
ZBTB16 promotion of osteogenesis is regulated by BRD4 binding with RPAP2. **a** ARS and ALP staining showing the effects of ZBTB16 knockdown and overexpression on MSC osteogenesis. Quantification of ARS and ALP are shown in the scatter plots. **b** Effects of ZBTB16 knockdown and overexpression on osteogenesis in vivo. HE, Masson and COL I immunohistochemistry staining of HA/TCP. Scatter plot showing Masson staining quantification. **c** BRD4 knockdown and overexpression and JQ1 treatment affected the mRNA expression of ZBTB16. **d** BRD4 knockdown and overexpression and JQ1 treatment affected the protein expression of ZBTB16 in MSCs. Scatter plots showing the relative protein abundance of BRD4 and ZBTB16. **e** Representative gel of BRD4-coimmunoprecipitated proteins stained with Coomassie blue to visualize the binding of BRD4 and RPAP2. **f** Diagram showing different BRD4 constructs. **g** Co-IP experiment showing the binding of different BRD4 constructs with RPAP2. **h** Effects of RPAP2 knockdown, RPAP2 knockdown and BRD4 overexpression, RPAP2 overexpression, BRD4 overexpression and BRD4 ΔET overexpression on the protein abundance of BRD4, ZBTB16, and RPAP2 in MSCs. Scatter plots showing the protein abundance of BRD4, ZBTB16 and RPAP2. The statistical data are represented as the means ± SEMs, *n* = 9, *P < 0.05, **P < 0.01, ***P < 0.005, ****P < 0.001

ZBTB16 siRNAs and overexpression plasmids were constructed and verified for efficacy (Fig. S2D–F). Inhibition of ZBTB16 expression in MSCs obviously decreased ARS and ALP staining intensity, and ZBTB16 overexpression led to an increase in staining intensity (Fig. 5a). The in vivo MSC osteogenesis assay showed disrupted bone formation in the ZBTB16 siRNA group and accelerated bone formation in the ZBTB16 OE group (Fig. 5b). These results confirmed that ZBTB16 promoted MSC osteogenesis.

BRD4 is one of the most important effectors of SEs in MSCs and participates in MSC osteogenesis via SEs (Fig. 2). Therefore, we postulated that SEs regulated *ZBTB16* expression via BRD4. Knockdown of BRD4 expression downregulated *ZBTB16* expression, and BRD4 overexpression increased *ZBTB16* expression in MSCs. The BRD4 inhibitor JQ1 downregulated ZBTB16 expression (Fig. 5c, d). To further investigate the mechanism of ZBTB16 expression regulated by SEs via BRD4, we performed coimmunoprecipitation (Co-IP) experiments and mass spectrometry of MSCs undergoing osteogenic differentiation for 7 days. The results showed that BRD4 bound to RPAP2, which was associated with the nuclear import of POL II (Fig. 5e and Table S5). BRD4 with deleted domains was constructed to confirm the RPAP2 binding sites in BRD4 (Fig. 5f). The BD1 and BD2 domains, which form the histone-binding pocket, and the carboxyterminal domain (CTD), which mediates the kinase activity of BRD4, showed no binding with RPAP2, but the extraterminal (ET) domain and the BRD4 ΔCTD construct that contained the ET domain showed clear binding (Fig. 5g). RPAP2 siRNAs and overexpression plasmids were constructed and verified (Fig. S2G–I). RPAP2 knockdown downregulated ZBTB16 expression and attenuated the promotion of ZBTB16 expression induced by BRD4 overexpression. Overexpression of BRD4 without the ET domain failed to upregulate ZBTB16 expression compared to the effect of full-length BRD4, and BRD4 overexpression did not alter RPAP2 expression (Fig. 5h). All of these results indicate that BRD4 regulation of ZBTB16 expression depends on the recruitment of RPAP2 by the SE effector BRD4.

### BRD4 navigates the translocation of the RPAP2-Pol II complex to SEs and drives *ZBTB16* transcription

**Fig. 6:**
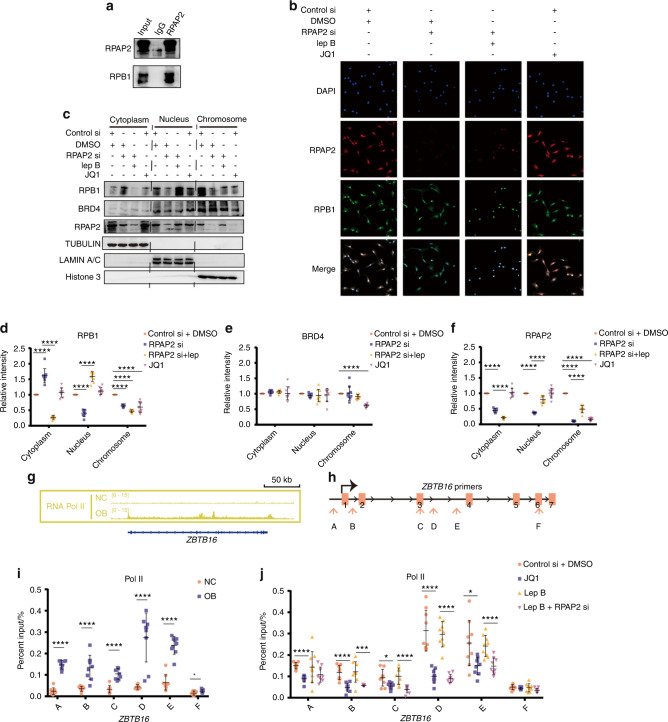
BRD4 navigates the translocation of the RPAP2-Pol II complex to SEs and drives *ZBTB16* transcription. **a** Co-IP experiment showing the binding of RPAP2 and RPB1. **b** Immunofluorescence showing the effects of RPAP2 knockdown, leptomycin B pretreatment prior to RPAP2 knockdown and JQ1 treatment on the subcellular distribution of RPAP2 and RPB1 in MSCs. **c**–**f** Western blot analysis of protein fractions showing the distribution of RPB1, BRD4 and RPAP2 in the cytoplasm, nucleus and chromatin. Tubulin, lamin A/C and histone 3 were the internal controls for proteins in the cytoplasm, nucleus and chromatin, respectively (**c**). Scatter plots showing the respective abundances of RPB1 (**d**), BRD4 (**e**) and RPAP2 (**f**) in different protein extracts. **g** ChIP-seq signal traces showing POL II binding to *ZBTB16* in NC or OB group MSCs. **h** Location of ChIP‒qPCR primers for *ZBTB16*. **i** ChIP‒qPCR analysis showing POL II occupancy on *ZBTB16* in NC and OB group MSCs. **j** ChIP‒qPCR analysis showing the effects of JQ1 treatment, leptomycin B and leptomycin B pretreatment prior to RPAP2 knockdown on POL II occupancy on *ZBTB16* in MSCs. The statistical data are represented as the means ± SEMs, *n* = 9, *P < 0.05, **P < 0.01, ***P < 0.005, ****P < 0.001

A previous study reported that RPAP2 was the nuclear importer of POL II,^20^ and we postulated that the transportation of POL II by RPAP2 participated in the regulation of *ZBTB16* expression. We confirmed the binding capacity of RPAP2 and POL II using Co-IP experiments (Fig. 6a). The subcellular location of RPAP2 and POL II, as shown by immunofluorescence experiments, revealed that RPAP2 knockdown inhibited the nuclear import of POL II. However, JQ1 did not affect the cellular distribution of POL II or RPAP2, which indicated that BRD4 did not participate in the transportation of POL II into the nucleus (Fig. 6b). We separated proteins into cytoplasmic, nuclear and chromatin-associated fractions. The POL II level was higher in the cytoplasm and lower in the nuclei and chromatin upon RPAP2 knockdown. Treatment of MSCs with 10 nmol·L^−1^ leptomycin B for 72 h, which inhibited nuclear export of proteins before RPAP2 knockdown, increased the POL II level in the nucleus but did not restore the decreased POL II level in the chromatin fraction. RPAP2 knockdown did not alter the distribution of BRD4, but the displacement of BRD4 from chromatin by JQ1 resulted in decreased expression of RPAP2 and POL II in the chromatin fraction (Fig. 6c–f). Taken together, these findings suggested that RPAP2 transported POL II into the nucleus, after which the RPAP2-POL II complex bound to BRD4 on SEs to initiate the transcription of targeted genes.

The POL II binding signal in *ZBTB16* increased significantly on the seventh day of osteogenic differentiation, which suggests the recruitment of POL II by *ZBTB16* SE (Fig. 6g). ChIP‒qPCR primers for *ZBTB16* were designed (Fig. 6h), and POL II ChIP‒qPCR of *ZBTB16* confirmed the increased binding of POL II to *ZBTB16* in the OB group (Fig. 6i). Consistent with these results, JQ1 treatment alone and leptomycin B treatment combined with RPAP2 knockdown inhibited the binding of POL II to *ZBTB16*, but treatment with leptomycin B alone did not produce this effect (Fig. 6j). In conclusion, after the nuclear transport of POL II by RPAP2, BRD4 guides the RPAP2-POL II complex to pinpoint SE-targeted *ZBTB16* to regulate its accurate and efficient expression and subsequent MSC osteogenic differentiation.

### BRD4 and RPAP2 promote *ZBTB16* transcriptional pause release and elongation by synergistically regulating RNA polymerase II subunit B1 (RPB1) CTD phosphorylation

**Fig. 7:**
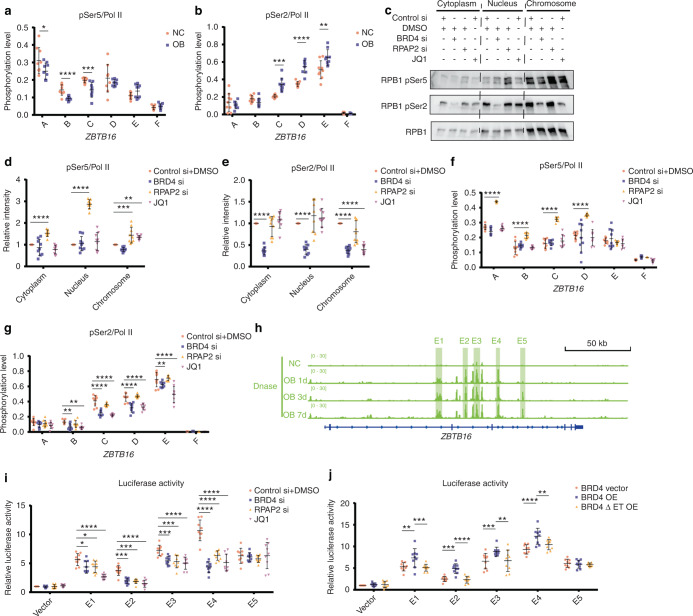
BRD4 and RPAP2 promote *ZBTB16* transcriptional pause release and elongation by synergistically regulating RPB1 CTD phosphorylation. **a** ChIP‒qPCR analysis showing the relative levels of pSer5 of the RPB1 CTD on *ZBTB16* in NC and OB group MSCs. **b** ChIP‒qPCR analysis showing the relative levels of pSer2 of the RPB1 CTD on *ZBTB16* in NC and OB group MSCs. **c**–**e** Western blot analysis of pSer5 and pSer2 in the cytoplasm, nucleus and chromatin (**c**). Scatter plots showing the relative levels of pSer5 (**d**) and pSer2 (**e**) in different protein extract fractions. **f** ChIP‒qPCR analysis showing the effects of RPAP2 knockdown, BRD4 knockdown and JQ1 treatment on the relative levels of pSer5 of the RPB1 CTD on *ZBTB16* in MSCs. **g** ChIP‒qPCR analysis showing the effects of RPAP2 knockdown, BRD4 knockdown and JQ1 treatment on the relative levels of pSer2 of the RPB1 CTD on *ZBTB16* in MSCs. **h** DNase-seq signal traces showing the accessibility of *ZBTB16* chromatin at different time points during osteogenic differentiation. Shadows show the constituent SE enhancers targeting *ZBTB16*. **i** Dual-luciferase reporter assays showing the effects of RPAP2 knockdown, BRD4 knockdown and JQ1 treatment on the transcriptional activity of the constituent SE enhancers targeting *ZBTB16* in MSCs. **j** Dual-luciferase reporter assays showing the effects of BRD4 and BRD4 ΔET overexpression on the transcriptional activity of the constituent SE enhancers targeting *ZBTB16* in MSCs. The statistical data are represented as the means ± SEMs, *n* = 9, *P < 0.05, **P < 0.01, ***P < 0.005, ****P < 0.001

Transcription is an intricate process that depends on the CTD phosphorylation level of the POL II subunit RPB1.^21^ Therefore, the relative levels of RPB1 CTD pSer5, as an indicator of transcriptional pause, and pSer2, as an indicator of transcriptional initiation, were measured using ChIP‒qPCR to evaluate *ZBTB16* transcription. The relative pSer5 levels in *ZBTB16* were generally decreased after osteogenic induction, and pSer2 levels were notably increased in MSCs undergoing osteogenic differentiation, which indicated that upregulated *ZBTB16* expression may be mediated by the phosphorylation of the RPB1 CTD (Fig. 7a, b). Recent studies have reported that RPAP2 and BRD4 are Ser5 phosphatases and Ser2 phosphokinases of the RPB1 CTD, respectively.^22,23^ Therefore, we investigated whether BRD4 and RPAP2 regulate the transcription of *ZBTB16* via POL II CTD phosphorylation. Knockdown of RPAP2 or BRD4 resulted in increased pSer5 levels and decreased pSer2 levels, respectively, in different cell compartments. Treatment of MSCs with JQ1 increased pSer5 levels and decreased pSer2 levels on chromatin without affecting the levels in the other compartments (Fig. 7c–e). These results revealed that BRD4 and RPAP2 synergistically initiated the elongation process of POL II by mediating the CTD phosphorylation states, which was also confirmed in the ChIP‒qPCR analyses of pSer5 and pSer2 levels in *ZBTB16* (Fig. 7f, g). SEs are generally composed of several component enhancers. Five component enhancers were identified in *ZBTB16* using DNase sequencing (DNase-seq), and luciferase reporter plasmids containing the component enhancer sequences were constructed (Fig. 7h). A dual-luciferase reporter assay showed that E1-4 promoted transcription, and knockdown of BRD4 and RPAP2 and JQ1 treatment disrupted the transcription-promoting effect of E1-4 (Fig. 7i). Overexpression of BRD4 enhanced the luciferase activity of E1-4, but the overexpression of a BRD4 mutant lacking the ET domain did not exert this effect (Fig. 7j).

To study the mechanism of ZBTB16 in MSC osteogenic regulation, we determined the effect of ZBTB16 on osteogenic TF expression. Knockdown of ZBTB16 specifically downregulated the transcription of *SP7* but not the other TFs (Fig. S5A). Knockdown of ZBTB16 downregulated the expression of SP7, and overexpression of ZBTB16 increased SP7 expression at the protein level. SP7 siRNAs were constructed and verified for efficacy (Fig. S2J, K). Knockdown of SP7 did not affect the expression of ZBTB16, which indicated that ZBTB16 acted at the early phase of osteogenesis and upstream of SP7 (Fig. S5B, C).

### Targeting *ZBTB16* protects against low bone mass and impaired bone repair in *Brd4*^fl/fl^*Prx1*-cre mice and OP models

**Fig. 8:**
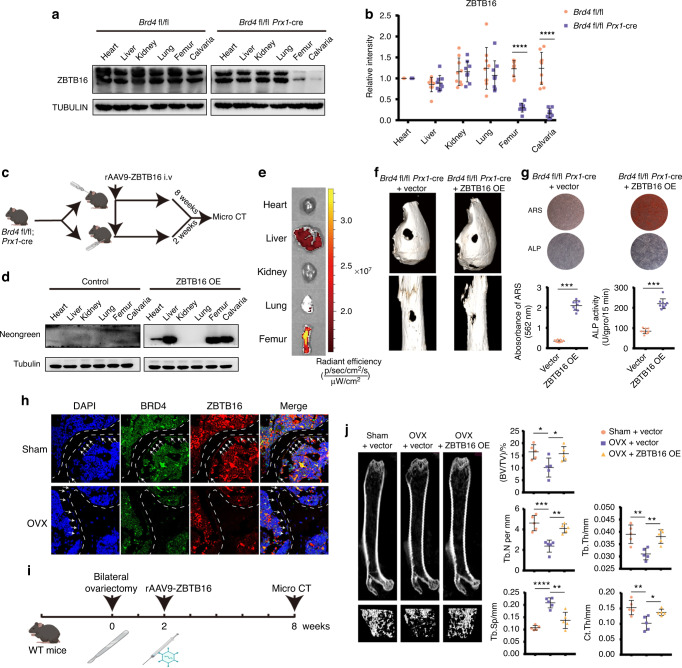
Targeting *ZBTB16* protects against low bone mass and impaired bone repair in *Brd4*^fl/fl^ Prx1-cre mice and OP models. **a** Immunoblot analysis of ZBTB16 protein expression in different organs of the *Brd4*^fl/fl^ and *Brd4*^fl/fl^ *Prx1*-cre mice. **b** Scatter plot showing the relative protein abundance of ZBTB16. **c** Diagram of rAAV9-ZBTB16 tail vein injection in mice with calvarial and femoral defects. **d** Immunoblot analysis showing the expression of neon green in different organs of the *Brd4*^fl/fl^ *Prx1*-cre mice injected with rAAV9-ZBTB16 or the vector control. **e** Fluorescence image of the *Brd4*^fl/fl^ *Prx1*-cre mice injected with rAAV9-ZBTB16. **f** Micro-CT analysis showing the calvarial and femoral defects of the *Brd4*^fl/fl^ *Prx1*-cre mice treated with rAAV9-ZBTB16 or the rAAV9 vector control injection. **g** ARS and ALP staining of osteogenic differentiating MSCs extracted from the *Brd4*^fl/fl^ *Prx1*-cre mice treated with rAAV9-ZBTB16 or rAAV9 vector control injection. Quantification of ARS and ALP is shown in the scatter plots. **h** Immunofluorescence showing the expression of BRD4 and ZBTB16 in femurs of the sham and OVX mice. **i** Workflow of rAAV9-ZBTB16 injection to treat the OVX mice. **j** Micro-CT analysis of the OVX mice treated with rAAV9-ZBTB16 or the rAAV9 vector control injection, and the trabecular bones were 3D reconstructed. Bone morphometric analysis was performed, and the parameters included BV/TV, Tb. Th, Tb. N, Tb. Sp and Ct. Th. The statistical data are represented as the means ± SEMs, *n* = 9 (*n* = 5 in **j**), *P < 0.05, **P < 0.01, ***P < 0.005, ****P < 0.001

*Brd4*^fl/fl^ *Prx1*-cre mice, but not control mice, showed selectively downregulated expression of BRD4 and ZBTB16 in femurs and calvarias (Fig. S6A). Quantification of BRD4 and ZBTB16 proteins in different tissues also showed collateral downregulation of ZBTB16 expression in femurs and calvarias (Fig. 8a, b). The previously developed bone-targeting recombinant adeno-associated virus 9 (rAAV9) was constructed for the in vivo bone-specific overexpression of ZBTB16.^24^ Calvarial and femoral defects were created in *Brd4*^fl/fl^ *Prx1*-cre mice and *Brd4*^fl/fl^ mice, and rAAV9-ZBTB16 was intravenously injected into the tails of these mice (Fig. 8c). The expression of neon green fluorescence, the marker for ZBTB16 AAV9 infection, was observed in calvarias and femurs, showing the bone-targeting features of rAAV9 (Fig. 8d). Additionally, the specific delivery of rAAV9 to the femurs was confirmed by fluorescence imaging of different organs (Fig. 8e). As shown in the micro-CT results, the bone healing capacity of the *Brd4*^fl/fl^ *Prx1*-cre mice was significantly enhanced after ZBTB16 overexpression (Fig. 8f and Fig. S6B). ARS and ALP assays showed that MSC osteogenesis in the *Brd4*^fl/fl^ *Prx1*-cre mice was increased after ZBTB16 overexpression (Fig. 8g). Femoral bone sections taken from ovariectomized (OVX) mice with postmenopausal OP showed decreased expression of BRD4 and ZBTB16 (Fig. 8h), which was similar to sections of femur head bones from healthy controls and patients with senescent OP (Fig. 4j). We intravenously injected rAAV9-ZBTB16 to treat the OVX osteoporotic mice (Fig. 8i). After treatment with rAAV9-ZBTB16, the OVX mice injected with rAAV9-ZBTB16 showed increased bone density and improvements in BV/TV, Tb. Th, Tb. N, Tb. Sp and Ct. Th compared to the OVX mice injected with the rAAV9 vector (Fig. 8j).

## Discussion

Skeletal homeostasis depends on the balance of bone reconstruction and resorption, and MSCs, as the major sources of osteogenic cells, are indispensable for bone homeostasis.^1^ The present study demonstrated that SEs were indispensable for MSC osteogenesis in vitro and in vivo, as well as for OP. Using integrative analyses of SE ChIP-seq and MSC/OP transcriptome sequencing, we identified *ZBTB16* as the most pivotal SE-regulated and OP-related gene in MSC osteogenesis. We further demonstrated that SEs mediated osteogenic differentiation via the navigation of POL II to *ZBTB16* through BRD4 and RPAP2. The synergistic effect of BRD4 phosphokinase and RPAP2 phosphatase activities mediated CTD phosphorylation of RPB1, which activated pause release and transcriptional elongation of *ZBTB16*. Bone-targeting ZBTB16 overexpression exerted a therapeutic effect on the decreased bone density and remodeling capacity of the *Brd4*^fl/fl^ *Prx1*-cre mice and OP models.

Many recent studies have investigated the mechanism of osteogenic cell lines in bone formation.^15,25,26^ However, two major critical issues must be addressed. These studies were performed using different cell lines under different experimental conditions. Therefore, contradictory and confusing results have been reported, and the common mechanism of osteogenesis in vivo is not clear. Many studies have focused on the post-transcriptional and translational regulatory mechanisms for the osteogenic differentiation of MSCs, including long noncoding RNA, m^6^A modification and protein modification.^27–29^ Studies on the upstream regulatory mechanism of osteogenesis at the transcriptional level are relatively rare. SEs are powerful cis-elements composed of multiple constituent enhancers, and the binding of TFs and cofactors present at excessively high densities leads to the highly efficient transcription of SE-targeted genes.^30^ As an upstream regulatory pattern, SEs are essential for key cell identity gene expression and sequential cell differentiation, including adipogenic, myogenic and trophoblastogenic cell differentiation.^5,31,32^ To investigate the effects of SEs on osteogenesis, we performed an integrated analysis of several SE ChIP-seq datasets in this study. These ChIP-seq data, generated by us and some other research groups, were obtained from different osteogenic cells with different SE markers,^14,15^ which contributed to the exploration of the most universal and upstream SEs in osteogenesis. OB-gained SEs in these ChIP-seq data were identified and enriched in osteogenesis-related terms, which indicated the important role of these OB-gained SEs in osteogenesis. We found that regulating the expression of BRD4, which is one of the most important markers and effectors of SEs, and the addition of the BRD4 inhibitor JQ1 negatively regulated MSC osteogenic differentiation. JQ1 decreased the SE signals of MSCs, as shown in the general CUT&Tag data heatmaps, which also confirmed the effect of SEs on osteogenesis.

To further confirm the effects of SEs on MSC osteogenesis in vivo, we generated *Brd4*^fl/fl^ Prx1-cre mice with CKO of BRD4 in MSCs, and decreased bone intensity and defective bone repair capacity were observed. Notably, the SE signals of MSCs from the *Brd4*^fl/fl^ *Prx1*-cre mice were significantly decreased in the general CUT&Tag data. Several studies have consistently reported that BRD4 positively regulates osteogenesis. Paradise demonstrated that BRD4 promoted osteoblast lineage commitment and maturation via Runx2. However, this research was performed using the mouse cell line MC3T3, and the mechanism may be different from that in human cells.^33^ Najafova reported that in human fetal osteoblasts, the colocalization of BRD4 and osteogenic TFs at the enhancers of osteogenic genes promotes osteogenesis. Although the ChIP-seq data of this study were included in our integrated analysis, one limitation must be noted: only one cell line was studied without in vivo experiments in this research.^15^ Our study determined the positive regulatory role of BRD4 and its mediated SEs on osteogenesis in vitro and in vivo and then clarified the most universal intrinsic mechanism and downstream targets, which filled the abovementioned research gaps. BRD4 CKO mice were also constructed by Paradise’s research group, who demonstrated that BRD4 was indispensable for chondrogenesis and endochondral ossification.^34^ Our study used BRD4 CKO mice and found that BRD4, as the most important marker and effector of SEs, contributed substantially to osteogenesis and intramembranous ossification.

Despite the critical role of SEs in osteogenesis, the detailed regulatory mechanism of SEs in osteogenesis is another key issue that needs clarification. Studies by different groups have reported a similar mechanism by which SEs exhibit their regulatory functions via their target genes.^35,36^ The present study found that only one common OB-gain SE located in the site of *ZBTB16* was identified in the five ChIP-seq datasets. Between the differentially expressed mRNAs and the OB-SE-targeting mRNAs, *ZBTB16* was one of the top 10 intersecting genes with the greatest upregulation and the lowest q values. These results indicated the critical and universal role of ZBTB16 in regulating osteogenesis. ZBTB16 belongs to the Kruppel-like zinc finger protein family, and the *ZBTB16*-encoded protein, as a TF with dual regulatory effects on transcription, participates extensively in cellular processes, including proliferation,^7^ differentiation^8^ and apoptosis.^9^ ZBTB16 regulates the decreased proliferation of osteoprogenitor cells to induce their maturation and promote osteoblast apoptosis during skeletal patterning,^11^ which indicates the contribution of ZBTB16 to osteogenesis. We demonstrated that the SE signals of *ZBTB16* were significantly enhanced after osteogenic differentiation. Consistent with a previous study,^37^ ZBTB16 expression increased during osteogenesis, which positively regulated MSC osteogenic differentiation in vitro and in vivo. Felthaus and colleagues showed that ZBTB16 promoted osteogenic marker expression in dental follicle cells independently of RUNX2.^13^ Onizuka demonstrated that ZBTB16 acted downstream of SP7 to increase the osteogenic ability of human periodontal ligament‐derived MSCs.^12^ However, we found that ZBTB16 was the upstream molecule of SP7, rather than its downstream molecule or other osteogenic TFs, to promote bone marrow-derived MSC osteogenesis. The reasons for these discrepancies may be the different cells and MSCs with different origins, which must be investigated in the future.

How SE regulates *ZBTB16* expression needs to be further clarified. Nucleosomes are composed of histones H2A, H2B, H3 and H4, and the DNA wraps around the globular domains. The structure of nucleosomes can be modified by the modification of protruding histones, which mediates chromatin accessibility. The high density of active histone modifications, such as H3K27ac, indicates accessible chromatin structures in SE regions, which show more efficient recruitment of TFs, cofactors and POL II for transcription. Because POL II is synthesized in the cytoplasm, the mechanisms by which POL II is transported from the cytoplasm to the nucleus and eventually to target genes on chromatin are worth investigating. A previous study reported that RPAP2 transports POL II into the nucleus.^20^ The present study showed that BRD4 bound to RPAP2 via the BRD4 ET domain, and knockdown of BRD4 or inhibition of BRD4 binding to histones reduced RPAP2 and POL II binding to chromatin. Combined treatment with leptomycin B and RPAP2 knockdown revealed no effect on POL II expression in the nucleus, but knockdown of RPAP2 expression inhibited BRD4 recruitment of POL II. We demonstrated that BRD4 on chromatin bound RPAP2 via the ET domain and navigated the RPAP2-POL II complex precisely to SE-targeted genes for rapid transcription during osteogenic differentiation.

Gene transcription starts at transcription start sites (TSSs) adjacent to promoters. However, ZBTB16-related SEs are located in its gene body rather than the TSSs. Therefore, another question remains: how does POL II recruitment to SEs accelerate *ZBTB16* transcription? Previous studies have shown that the mediator coactivator complex subunit MED1 regulates the conformational changes of chromatin to enable the direct interaction of promoters and enhancers. Disruption of MED1 hindered chromatin conformational changes and subsequently influenced POL II binding to promoters in cardiomyocytes, which inhibited transcription.^38^ The intrinsically disordered regions of MED1 promote the formation of phase-separation condensates composed of SEs. The condensates accumulate very high concentrations of BRD4 and POL II to drive efficient transcription within the region.^16^ Although further investigation is needed, we speculate that chromatin remodeling and looping are intrinsic mechanisms by which SEs share or move POL II to promoters, and the SE-formed phase-separation condensates create separate spaces for robust transcription.

After the recruitment of POL II to the target genes, instead of initiating transcription immediately, POL II binds to DNA and pauses after transcribing 20–120 nucleotides downstream of a TSS, a process named transcriptional pause.^39^ Transcriptional pause ensures rapid and synchronous gene transcription,^40^ including osteogenic identity gene transcription during osteogenic differentiation. The CTD phosphorylation sites of the POL II subunit RPB1 are composed of multiple tandemly repeated heptapeptides with the consensus sequence Tyr-Ser-Pro-Thr-Ser-Pro-Ser (Y_1_S_2_P_3_T_4_S_5_P_6_S_7_), which controls the state of POL II during transcription.^21^

Phosphorylation of Ser5 recruits the capping enzyme of mRNA during transcription, and the capping of mRNA maintains the POL II transcriptional pause. Phosphorylation of Ser2 induces the exit of transcriptional pause and launches the transcriptional elongation of POL II.^41^ During osteogenic differentiation, the levels of pSer5 in *ZBTB16* decreased and pSer2 increased in our study, which indicated the transition from transcriptional pause to transcriptional elongation. Previous studies have shown that RPAP2 is a Ser5 phosphatase,^42^ and BRD4 is a Ser2 phosphokinase that acts on the RPB1 CTD.^23^ We performed ChIP‒qPCR and dual-luciferase reporter assays and confirmed the synergistic regulation of RPB1 CTD phosphorylation and activation of transcription elongation by BRD4 and RPAP2 during osteogenic differentiation to reveal the intrinsic SE mechanism for enhancing *ZBTB16* transcription during osteogenic differentiation. Therefore, BRD4 binds to SEs at histone-binding pockets composed of BD1 and BD2 domains, recruits the RPAP2-RPB1 complex to induce transcriptional pause on SE-targeted genes via the BRD4 ET domain, and regulates the Ser2 phosphorylation of RPB1 CTD to release the transcriptional pause and initiate transcriptional elongation.

As a systemic disease, osteoporosis causes decreased bone density and microdamage to bone structures, subsequently leading to a high risk of bone fractures.^43^ Elucidating the pathogenesis and then developing novel therapies for OP are critical areas in this field.^44^ Dysfunction of MSCs, the major origin of osteoblasts, in osteogenesis was demonstrated to contribute to OP pathogenesis, but the detailed mechanism still needs further investigation.^45,46^ In our study, we showed that BRD4 expression was downregulated in OP-MSCs, and BRD4-CKO mice exhibited a disease phenotype similar to OP. In addition, the expression of the most common SE target, ZBTB16, was decreased in OP-MSCs. The above results indicate the critical role of SE-targeting ZBTB16 in OP pathogenesis and suggest its potential for OP treatment. AAV is one of the most extensively investigated gene therapy vehicles.^47^ Recently, Yeon-Suk Yang and colleagues grafted the bone-targeting peptide motif (Asp-Ser-Ser)6 to the AAV9-VP2 capsid protein, enabling the bone-specific overexpression of the targeted genes.^24^ Herein, we found that ZBTB16 overexpression induced by bone-targeting rAAV9 successfully reversed the low bone mass in the *Brd4*^fl/fl^ *Prx1*-cre mice. The above results verified that impaired skeletal balance in the *Brd4*^fl/fl^ *Prx1*-cre mice was a consequence of ZBTB16 expression downregulation after BRD4 depletion and emphasized the important role of SE-targeting ZBTB16 in OP therapy. Furthermore, the bone-targeted overexpression of ZBTB16 also reversed OP in the OVX mice. Although this conclusion must be confirmed, ZBTB16 overexpression induced by bone-targeting rAAV9 may be a safe, effective and highly specific treatment for patients with OP.

In conclusion, we clarified the intrinsic mechanism by which SEs precisely and efficiently regulate *ZBTB16* transcription to orchestrate osteogenic progression, which may provide novel therapeutic targets for OP. Limitations remain in our study, such as the unclear mechanism of ZBTB16 for SP7 regulation and the absence of conditional knock-in ZBTB16 mice, which should be addressed in the future.

## Materials and methods

### Study approval

This study was approved by the Ethics Committee of the Eighth Affiliated Hospital, Sun Yat-Sen University, Guangzhou, China. Seventeen OP patients and twenty-one control subjects (NCs) without OP who needed spine surgery were recruited. The diagnostic criterion of OP patients was a BMD T score less than -2 at the lumbar spine. After signed informed consent was provided, bone tissue was acquired during the surgery, and bone marrow punctures were performed to extract MSCs. See Table S6 for information on the recruited subjects. The murine experiments were approved by The Institutional Animal Care and Use Committee of Sun Yat-Sen University, Guangzhou, China.

### MSC isolation and culture

After bone marrow punctures of NCs and OP patients, density gradient centrifugation at 12 000 r·min^−1^ for 30 min (Invitrogen) was used to extract MSCs from the bone marrow. The extracted MSCs were cultured in Dulbecco’s modified Eagle’s medium (DMEM, Gibco) containing 10% fetal bovine serum (FBS, Hangzhou Sijiqing Biological Engineering Material Company, Limited).

For mouse MSC isolation, femurs and tibias were collected and cut into pieces. After filtration through a 40 µm cell strainer (BD, Cat. No. 352340), the filtrate was resuspended using the MesenCult Expansion Kit (Stemcell, Cat. No. 05513). The MSCs adhered to the flask, and nonadherent cells were removed after five days.

For osteogenic induction, MSCs were cultured in osteogenic medium consisting of 10% FBS DMEM containing 100 IU·mL^−1^ penicillin, 100 IU·mL^−1^ streptomycin, 0.1 μmol·L^−1^ dexamethasone, 10 mmol·L^−1^ β-glycerol phosphate, and 50 μmol·L^−1^ ascorbic acid (Sigma‒Aldrich). The osteogenic medium was changed every three days. JQ1 (ApexBio, Cat. No. A1910) was used to treat MSCs.

### Plasmid and siRNA infection

BRD4, ZBTB16 and RPAP2 siRNAs were purchased from GenePharma (Shanghai, China) (Fig. S2).

The BRD4, ZBTB16, RPAP2, BRD4-FL-FLAG, BRD4-BD1-FLAG, BRD4-BD2-FLAG, BRD4-ET-FLAG, BRD4-CTD-FLAG, BRD4-ΔET-FLAG, and BRD4-ΔCTD-FLAG overexpression plasmids and the dual-luciferase reporter plasmids E1-5 were constructed by ObiO Technology (Shanghai) Corp., Ltd. See Table S7 for detailed information.

### Animal models

Eight-week-old wild-type C57BL/6 mice and BALB/c-nu/nu mice were purchased from the Laboratory Animal Center of Sun Yat-Sen University.

#### *Brd4*^fl/fl^*Prx1*-cre CKO mice

C57BL/6 Prx1-cre mice were purchased from the Jackson Laboratory. C57BL/6 *Brd4*^fl/fl^ transgenic mice were purchased from GemPharmatech to construct *Brd4*^fl/fl^ *Prx1*-cre CKO mice. The following PCR primers were used for genotyping *Brd4*^fl/fl^: 5’ arm primers, forward, GGATTTCCATAGGTCTTCATTTGCT, and 5’ arm primers reverse, CAGAGGAGAGCATGAAGATATGTTCC. Only the 250-bp DNA bands will be detected in wild-type mice, and only the 355-bp DNA bands will be detected in homozygous *Brd4*^fl/fl^ mice. Both 250-bp and 355-bp PCR DNA bands will be detected in heterozygous mice (*Brd4*^fl/-^). PCR primers for the Cre sequence were used to detect the *Prx1*-cre transgene in *Prx1*-Cre mice: *Prx1*-cre forward, GCTCTGATGTTGGCAAAGGGGT, and *Prx1*-cre reverse, AACATCTTCAGGTTCTGCGGG.

#### Osteogenic induction in vivo

MSCs in the third passage were induced for osteogenic differentiation. After seven days of osteogenic induction, MSCs (5 × 10^5^) were collected and transplanted on hydroxyapatite (HA)/tricalcium phosphate (TCP) (Zimmer) for 24 h. The MSC-loaded HA/TCP grafts were transplanted into the subcutaneous dorsal space of eight-week-old BALB/c nu/nu mice, which were then treated with osteogenic medium containing DMSO or JQ1 via local injection every three days. The mice were euthanized via cervical dislocation eight weeks after implantation, and the grafts were collected for hematoxylin and eosin (HE) and Masson staining and histochemistry analyses.

#### Calvarial and femoral bone defects in mice

An electric bone drill was used to create bone defects in the calvarial bone and femur of eight-week-old mice. Before the procedures, the mice were sacrificed and disinfected. The skin was incised, and subcutaneous tissue was separated to expose the calvarial bone and femur. An electric bone drill with a 2.5-mm sterilized drill bit was used to create calvarial bone defects, and a 1.0-mm drill bit was used to create femur defects. Whole skulls were collected eight weeks later, and femurs were collected two weeks later for micro-CT analysis.

#### OVX mice

Eight-week-old female mice were subjected to bilateral ovariectomy, and other mice underwent sham surgery. After two months of surgery, the mice were sacrificed for subsequent experiments.

#### Bone-targeting ZBTB16 overexpression

Bone-targeting rAAV9-ZBTB16 was designed and constructed as previously described.^24^ The DNA sequence encoding the bone-specific peptide motif DSS (Asp-Ser-Ser)_6_ was inserted into the AAV9 capsid protein VP2 to build the rAAV9-ZBTB16 bone-targeting overexpression vectors.

### ARS and ALP assays

MSCs were rinsed twice with phosphate-buffered saline (PBS), and then, 4% paraformaldehyde (PFA) was used to fix MSCs for 30 min.

For ARS staining, MSCs were stained with 1% ARS (pH 4.2) (Solarbio, Cat. No. G8550) for 15 min at room temperature. After removal of the nonspecific stains with PBS, the images of stained MSCs were captured. Cetylpyridinium chloride monohydrate (10%, Sigma–Aldrich, Cat. No. 8400080100) was used to extract ARS staining for quantification.

For ALP staining, MSCs were stained using a 5-bromo-4-chloro-3-indolyl phosphate (BCIP)/nitro blue tetrazolium (NBT) alkaline phosphatase kit (Beyotime Institute of Biotechnology, Cat. No. C3206). For the ALP activity assay, MSCs were lysed in RIPA buffer (Sigma‒Aldrich, Cat. No. R0278). ALP activity was detected using ALP activity kits (Nanjing Jiancheng Biotech, Nanjing, China, Cat. No. A059-2), and ALP activity was quantified at 405 nm using a microplate reader.

### Histological staining

Bone tissues and the MSC-loaded HA/TCP were collected and fixed in 4% PFA overnight at 4 °C. The HA/TCP was decalcified in 20% EDTA for subsequent paraffin embedding. Slides were stained with HE (Boster, Cat. No. AR1180) and Masson stain (Solarbio, Cat. No. G1340-100). Immunohistochemistry was performed with an anti-COL1 antibody (Abcam, Cat. No. ab34710).

### Immunofluorescence

The bone of NCs and OP patients and the calvarial and femoral bones of mice were collected for immunofluorescence analysis. Bone tissue sections were deparaffinized and rehydrated. Citrate buffer (pH 6.0) at a concentration of 10 mmol·L^−1^ was used for antigen retrieval. Sections were immersed in citrate buffer and microwaved for 15 min. Cultured MSCs were fixed with 4% PFA for 15 min before immunofluorescence. After permeabilization with 0.5% Triton X-100 for 20 min, bone sections or MSCs were blocked with 10% FBS in PBS for 1 h. After incubation with anti-ZBTB16 (Abcam, Cat. No. ab104854), anti-BRD4 (Cell Signaling Technology, Cat. No. 13440S), anti-POL II CTD (Santa Cruz, Cat. No. sc-47701) or anti-RPAP2 (Proteintech, Cat. No. 17401-1-AP) primary antibodies overnight at 4 °C, the samples were incubated with the following secondary antibodies for 1 h at room temperature: anti-mouse Alexa 488 (Cell Signaling Technology, Cat. No. 4408) and anti-rabbit Alexa 555 (Cell Signaling Technology, Cat. No. 4413). We used DAPI antifade mounting medium (Beyotime, Cat. No. P0131) for mounting. Images were captured using a Zeiss LSM 880 confocal microscope.

### RNA isolation and qRT‒PCR analysis

RNAiso Plus (TaKaRa, Cat. No. 9109) was used to extract RNA from MSCs, and the PrimeScript™ RT reagent kit (TaKaRa, Cat. No. RR036A) was used to reverse transcribe the isolated RNA into cDNA. SYBR Premix Ex Taq™ (TaKaRa, Cat. No. RR420A) was used to perform qRT‒PCR in a LightCycler R480 PCR system (Roche). GAPDH was used as the reference gene to normalize the expression of the target genes. Each qRT‒PCR analysis was performed in triplicate. See Table S8 for the primer sequences.

### Co-IP

Cell lysis buffer for Western blot and IP (Beyotime, Cat. No. P0013) were used to extract proteins from MSCs. A Dynabeads™ protein G immunoprecipitation kit (Invitrogen, Cat. No. 10007D) was used for Co-IP. Dynabeads were resuspended and placed on a magnet to remove the supernatant and then rotationally incubated with Ab Binding & Washing Buffer containing an anti-Flag antibody (Cell Signaling Technology, Cat. No. 14793) or an anti-RPAP2 antibody (Proteintech, Cat. No. 17401-1-AP) for 10 min at room temperature. After removal of the supernatant, the Dynabeads-antibody complex was washed with Ab Binding & Washing Buffer, and MSC lysates were added to the Dynabeads-antibody mixture and rotationally incubated for 10 min at room temperature. After removal of the supernatant, the Dynabeads-antibody complex mixture was washed with washing buffer, and the proteins were separated using standard SDS–polyacrylamide gel electrophoresis (SDS‒PAGE system).

### Mass spectrometry

Protein samples were mixed with 5X loading buffer and boiled for 5 min. After separation in a 10% SDS‒PAGE gel, Coomassie Blue staining (Solarbio, Cat. No. P1305) was used to visualize the protein. LC‒MS/MS analysis was performed in a Q Exactive mass spectrometer (Thermo Scientific) coupled to Easy nLC (Proxeon Biosystems, now Thermo Fisher Scientific) for 120 min. The raw MS data for each sample were combined and searched using MaxQuant (v1.5.3.17) software.

### Protein extraction and Western blot

Cells and crushed tissues were lysed in ice-cold RIPA buffer (Sigma–Aldrich, Cat. No. R0278), followed by centrifugation at 12 000 r·min^−1^ at 4 °C for 30 min to extract whole-cell proteins. NE-PER™ nuclear and cytoplasmic extraction reagents (Invitrogen, Cat. No. 78833) were used for protein fractionation. A chromatin extraction kit (Abcam, Cat. No. ab117152) was used to remove chromatin proteins from the intact nucleic pellets obtained with the nuclear extraction reagent in the previous step. Proteins were separated on 6% or 10% SDS–PAGE gels and transferred to PVDF membranes (Merck Millipore, Cat. No. IPVH00010). The membranes were blocked with 5% nonfat milk dissolved in Tris-buffered saline with Tween 20 (TBST) and incubated with primary antibodies overnight at 4 °C. PVDF membranes were incubated with horseradish peroxidase (HRP)-conjugated anti-mouse antibody (Cell Signaling Technology, Cat. No. 7076) or HRP-conjugated anti-rabbit antibody (Cell Signaling Technology Cat. No. 7074) for 1 h at room temperature, after which chemiluminescence reagents (Millipore, Cat. No. WBKLS0500) was used to determine the protein levels on the PVDF membranes. The following primary antibodies were used: anti-BRD4 (Cell Signaling Technology, Cat. No. 13440S), anti-COL1 (Abcam, Cat. No. ab34710), anti-GAPDH (Cell Signaling Technology, Cat. No. 5174S), anti-ZBTB16 (Abcam, Cat. No. ab39354), anti-RPAP2 (Proteintech, Cat. No. 17401-1-AP), anti-Flag (Cell Signaling Technology, Cat. No. 14793), anti-POL II CTD (Abcam, Cat. No. ab26721), anti-POL II CTD (pSer5) (Abcam, Cat. No. ab26721), anti-POL II CTD (pSer2) (Abcam, Cat. No. ab193468), anti-β-tubulin (Cell Signaling Technology, Cat. No. 2128), anti-histone H3 (Abcam, Cat. No. ab10799), and anti-lamin A/C (Cell Signaling Technology, Cat. No. 4777).

### ChIP, qPCR and sequencing

An EpiQuik^TM^ chromatin immunoprecipitation kit (Epibiotek) was used for ChIP experiments. Approximately 1 × 10^7^ MSCs were harvested, crosslinked with 1% formaldehyde for 10 min and quenched with 0.125 mol·L^−1^ glycine for 5 min. Then, the cells were lysed with 1 mL of lysis buffer followed by rotational incubation for 30 min at 4 °C. The lysates were centrifuged at 2 400 × *g* for 10 min at 4 °C to isolate nuclei. Digestion buffer was used to enzymatically digest the chromatin into fragments between 200 bp and 500 bp. These fragments were obtained in a tube at 37 °C. The chromatin fragments were centrifuged at 18 000 × *g* for 10 min at 4 °C. The supernatant was transferred to a ChIP reaction mix containing protein A/G magnetic beads, ChIP IP buffer, an anti-H3K27ac antibody (07-360, Sigma–Aldrich), an anti-BRD4 antibody (A301-985A, Bethyl), an anti-POL II CTD antibody (Abcam, Cat. No. ab26721), an anti-POL II CTD antibody (pSer5) (Abcam, Cat. No. ab26721) or an anti-POL II CTD antibody (pSer2) (Abcam, Cat. No. ab193468), and protease inhibitor cocktail was added. After rotational incubation overnight at 4 °C, the protein A/G magnetic beads were collected using a magnet. Chromatin was eluted in reverse crosslinking buffer and incubated at 65 °C for 3 h. The ChIP DNA was treated with RNase A and protease K at 37 °C for 30 min and purified using the phenol–chloroform method.

ChIP DNA was subjected to ChIP‒qPCR analysis or processed for library preparation using a QIAseq Ultralow Input Library Kit (Qiagen). The ChIP‒qPCR primers are listed in Table S8.

### ChIP-seq data analysis

TrimGalore (v0.6.6) was used to filter low-quality reads and trim adaptors using the following parameters --phred33 -q 20 -stringency 3. Bowtie2 (v2.5.1) was used to map the filtered clean reads to the hg38 genome using default parameters. MACS2 (v2.1.1) was used to call peaks with the following parameters: DNase-seq: --nomodel --shift -75 --extsize 150 -g hs, ChIP-seq: -m 5 50 -p 1e-5 -g hs. DeepTools (v2.3.6.0) was used to normalize the ChIP-seq and DNase-seq data in reads per kilobase per million (RPKM) using the bamCoverage command and to plot the heatmaps of ChIP-seq and DNase-seq data using the computeMatrix and plotHeatmap commands.

Enhancers were predicted from the H3K27ac, BRD4 and MED1 peaks. Enhancers within the 12.5 kb region were stitched together, and stitched enhancers were ranked according to peak signal to identify SEs using ROSE algorithm version 2 developed by the Young laboratory.^48^ Stitched enhancers with signals higher than the signals with a slope of 1 on the intensity distribution plot were considered SEs and others were TEs. Script annotatePeaks.pl in Homer (v4.11.1)^49^ was used to annotate TEs and SEs with default parameters.

The data of TERT4-MSC DNase-seq and H3K27ac and MED1 ChIP-seq data were downloaded from GSE113253, and hFOB1.19 H3K27ac, BRD4 and RNA POL II ChIP-seq data were downloaded from GSE82295. We generated hBMMSC H3K27ac ChIP-seq data.

### RNA-seq library preparation and sequencing

RNA was extracted as described above. RNA purified by oligo(dT)-attached magnetic beads was fragmented. Random hexamer-primed reverse transcription was used to generate first-strand cDNA, and second-strand cDNA was synthesized. An A-Tailing Mix and RNA Index Adaptors were used for end repair. PCR-amplified cDNA fragments were purified with AMPure XP Beads, and EB solution was added to dissolve the products. The double-stranded PCR products were heated, denatured and circularized to generate the final library. The final library was amplified with phi29 to generate DNA nanoballs (DNBs) with more than 300 copies of a molecule. The DNBs were loaded into a patterned nanoarray, and single-end 50-base reads were generated using a BGISeq500 platform (BGI-Shenzhen, China).

### RNA-seq data analysis

SOAPnuke (v1.5.2)^50^ was used to filter raw data to remove adaptors and low-quality reads (base rate higher than 20% or an unknown base (‘N’ base) rate higher than 5%). The clean reads were mapped to the hg38 genome for quality control using HISAT2 (v2.0.4).^51^ Bowtie2 (v2.5.1)^52^ was used to align the clean reads to the hg38 reference, and RSEM (v1.2.12)^53^ was used to calculate the gene expression. A heatmap was drawn using pheatmap (v1.0.8) (https://CRAN.R-project.org/package=pheatmap). DESeq2 (v1.4.5)^54^ was used for differential expression analysis with a cutoff |log2fc| ≥ 1 and Q value ≤ 0.05.

The R package clusterProfiler (v3.11) was used to perform Kyoto Encyclopedia of Genes and Genomes (KEGG, https://www.kegg.jp/) and GO (http://www.geneontology.org/) enrichment analyses of DEGs. The cutoff threshold of significantly enriched terms was a Q value ≤ 0.05 using the Bonferroni method. GSEA was performed using OmicStudio tools at https://www.omicstudio.cn/tool.

### CUT&Tag assay

NovoNGS® CUT&Tag 2.0 A High-Sensitivity Kit (for Illumina®) (Novoprotein Scientific, Inc., Cat# N259-YH01-01A) was used to perform the CUT&Tag assay. Briefly, cells were harvested and enriched by ConA magnetic beads. A total of 50 000 cells were resuspended and washed twice with 100 μL of Dig-wash Buffer. The samples were incubated with primary BRD4 antibody (1:100, 4 °C, 18 h) and secondary antibody (1:200, 25 °C, 1 h). After incubation, the beads were washed three times in Dig-Hisalt Buffer. Cells were incubated with the protein A-Tn5 transposome at 25 °C for 1 h and washed three times in Dig-Hisalt buffer. The cells were resuspended in 50 μL of tagmentation buffer, incubated at 37 °C for 1 h and then terminated with 1 μL of 10% SDS at 55 °C for 10 min. Phenol chloroform was used to extract the DNA fragments.

### CUT&Tag sequencing and analysis

The libraries were used for sequencing on an Illumina NovaSeq 6000 platform at Novogene Science and Technology Co., Ltd. (Beijing, China), which generated PE150 sequencing data. TrimGalore (v0.6.6) was used to filter the sequencing adaptors and low-quality reads with the parameters -q 20 --phred33 --stringency 3. Bowtie2 (v2.5.1)^52^ was used to map clean reads to the hg38 genome with the default parameters. MACS2 (v.2.1.1)^55^ was used to call peaks with the parameter -q 0.05 --call-summits --nomodel--shift -100 --extsize 200 --keep-dup all. The computeMatrix and plotHeatmap commands in deepTools (v2.3.6.0)^56^ were used to plot heatmaps of the CUT&Tag data.

### Dual-luciferase reporter assay

MSCs were cotransfected with a pRLTK plasmid and a pGL4.26-basic or pGL4.26 plasmid carrying the respective constituent SE enhancer targeting ZBTB16 with Lipofectamine 3000 (Invitrogen, Cat. No. L3000015). Luciferase activity was measured using the Dual-Glo luciferase assay system (Promega, Cat. No. E1910) 48 h post-transfection. Firefly luciferase activity was normalized to Renilla luciferase to control for the cell number and transfection efficiency.

### Micro-CT scanning

For evaluation of bone structures, a micro-CT assay was performed using the Inveon MM system (Siemens). Images were acquired at each of 360 rotational steps with a pixel size of 8.82 lm, a voltage of 80 kV, a current of 500 lA and an exposure time of 1 500 ms. The parameters BV/TV, Tb. Th, Tb. N and Ct. Th and Tb. Sp were calculated using an Inveon Research Workplace (Siemens).

### Statistics

GraphPad Prism (v7.00) was used for statistical analyses. For comparisons between two groups, unpaired Student’s t tests were used. When comparing means between three or more groups, we used one-way analysis of variance (ANOVA) with Bonferroni’s multiple comparison tests. Data are presented as the means ± SEMs. We indicated significance as *P < 0.05, **P < 0.01, ***P < 0.005 and ****P < 0.001.

## Supplementary information

The iThenticate duplicate report of the manuscript

Graphic abstract

Supplementary Figure 1

Supplementary Figure 2

Supplementary Figure 3

Supplementary Figure 4

Supplementary Figure 5

Supplementary Figure 6

Supplementary Table 1

Supplementary Table 2

Supplementary Table 3

Supplementary Table 4

Supplementary Table 5

Supplementary Table 6

Supplementary Table 7

Supplementary Table 8

## Data Availability

ChIP-seq, RNA-seq and CUT&Tag data of BMMSCs have been deposited in the NCBI Gene Expression Omnibus (GEO) repository and are available in GSE192963. Mass spectrum data have been uploaded to the iProX database, and the ProteomeXchange ID is PXD034615. The link to the data is https://www.iprox.cn/page/PSV023.html;?url=1655430492356dXCk. The raw data from previous studies were obtained from the GEO repository, TERT4-MSC DNase-seq and H3K27ac and MED1 ChIP-seq in GSE113253 and hFOB1.19 H3K27ac, BRD4 and RNA POL II ChIP-seq data in GSE82295 The raw RNA-seq data of OP vs. NC were obtained from the SRA repository in PRJNA763497.
