# Author Correction: Super enhancers targeting ZBTB16 in osteogenesis protect against osteoporosis

**DOI:** 10.1038/s41413-023-00275-8

**Published:** 2023-06-26

**Authors:** Wenhui Yu, Zhongyu Xie, Jinteng Li, Jiajie Lin, Zepeng Su, Yunshu Che, Feng Ye, Zhaoqiang Zhang, Peitao Xu, Yipeng Zeng, Xiaojun Xu, Zhikun Li, Pei Feng, Rujia Mi, Yanfeng Wu, Huiyong Shen

**Affiliations:** 1 Department of Orthopedics, The Eighth Affiliated Hospital, Sun Yat-sen University, 518003 Shenzhen, PR China; 2 Shenzhen Key Laboratory of Ankylosing Spondylitis, 518003 Shenzhen, PR China; 3 Department of Orthopedics, Sun Yat-sen Memorial Hospital, Sun Yat-sen University, 510120 Guangzhou, PR China; 4 Center for Biotherapy, The Eighth Affiliated Hospital, Sun Yat-sen University, 518003 Shenzhen, PR China

**Keywords:** Osteoporosis, Bone, Pathogenesis

Correction to: *Bone Res* 10.1038/s41413-023-00267-8, published online 7 June 2023

Following publication of this article,^1^ it is noticed that the terms “immortal TERT4-MSCs” and “hFOB1.19 cells” were switched in Fig. 1, Fig. 4 and Supplementary Fig. 1.

The correct Fig. 1 should read:
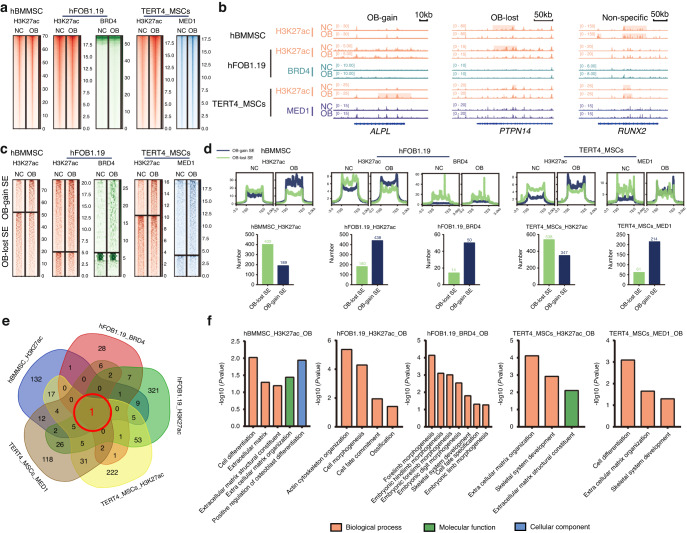


**Fig. 1** SE profile analysis and identification of critical OB-gain SEs. **a** ChIP-seq profile heatmaps showing H3K27ac abundance in hBMMSCs, H3K27ac and BRD4 abundance in hFOB1.19 cells and H3K27ac and MED1 abundance in immortal TERT4-MSCs. **b** Example signal traces of OB-gain, OB-lost and nonspecific SEs. The shadows indicate SE regions. **c** ChIP-seq profile heatmaps of the SEs identified by H3K27ac in hBMMSCs, H3K27ac and BRD4 in hFOB1.19 cells and H3K27ac and MED1 in immortal TERT4-MSCs. **d** The average SE signal levels are shown in line plots, and the numbers of OB-lost and OB-gain SEs are shown in histograms. **e** Venn diagram showing the intersecting OB-gain SEs from different datasets. **f** GO analyses of OB-gain SEs from different datasets

The correct fig. 4 should read:
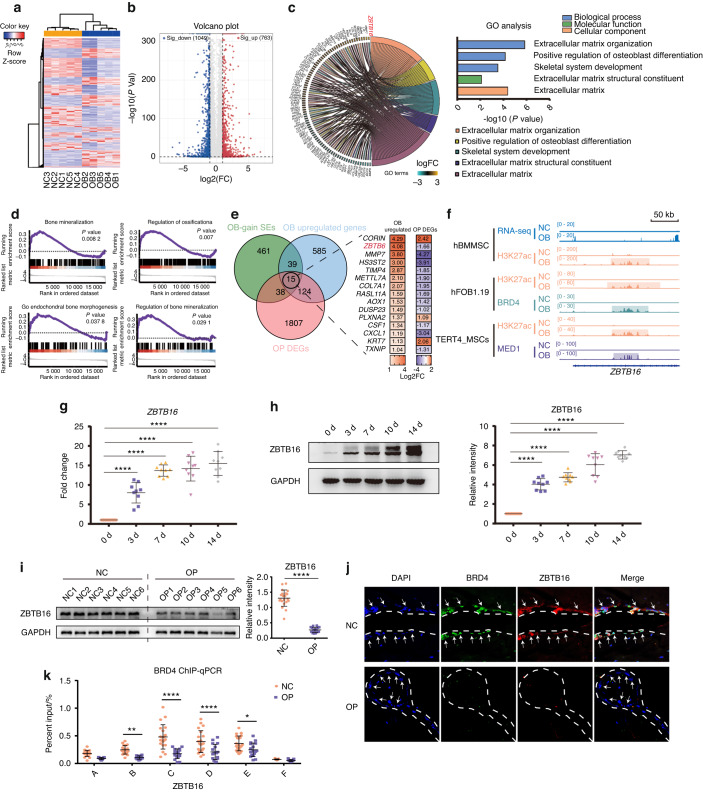


The correct Supplementary Fig. 1 should read:
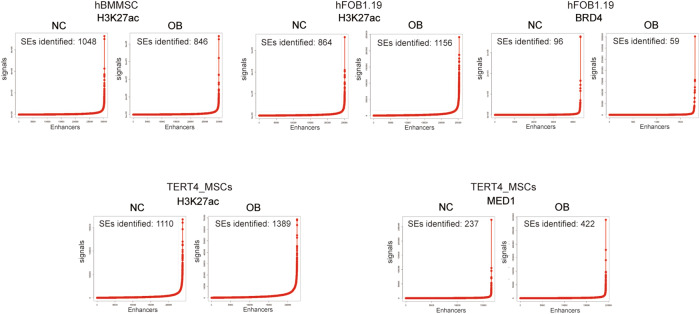


The affected texts mentioning the two terms have been corrected in the article.

The original article^1^ was updated.
