# Supplementary figures and images for: Super enhancers targeting ZBTB16 in osteogenesis protect against osteoporosis
Source: Bone Res. 2023 Jun 7;11:30. doi: 10.1038/s41413-023-00267-8 (PMC10244438; doi:10.1038/s41413-023-00267-8)

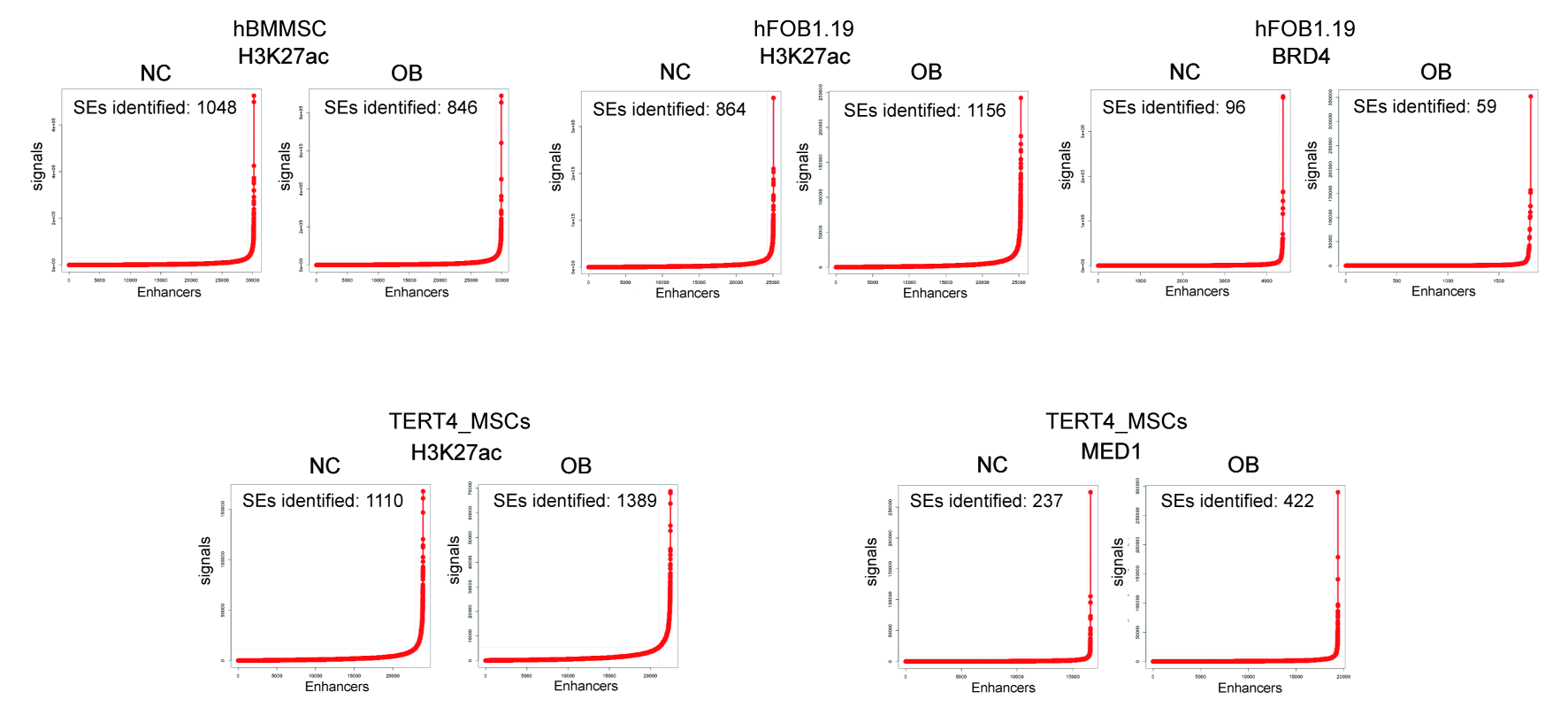

Supplement: Supplementary file 2 — Graphic abstract [file 41413_2023_267_MOESM2_ESM.tif]

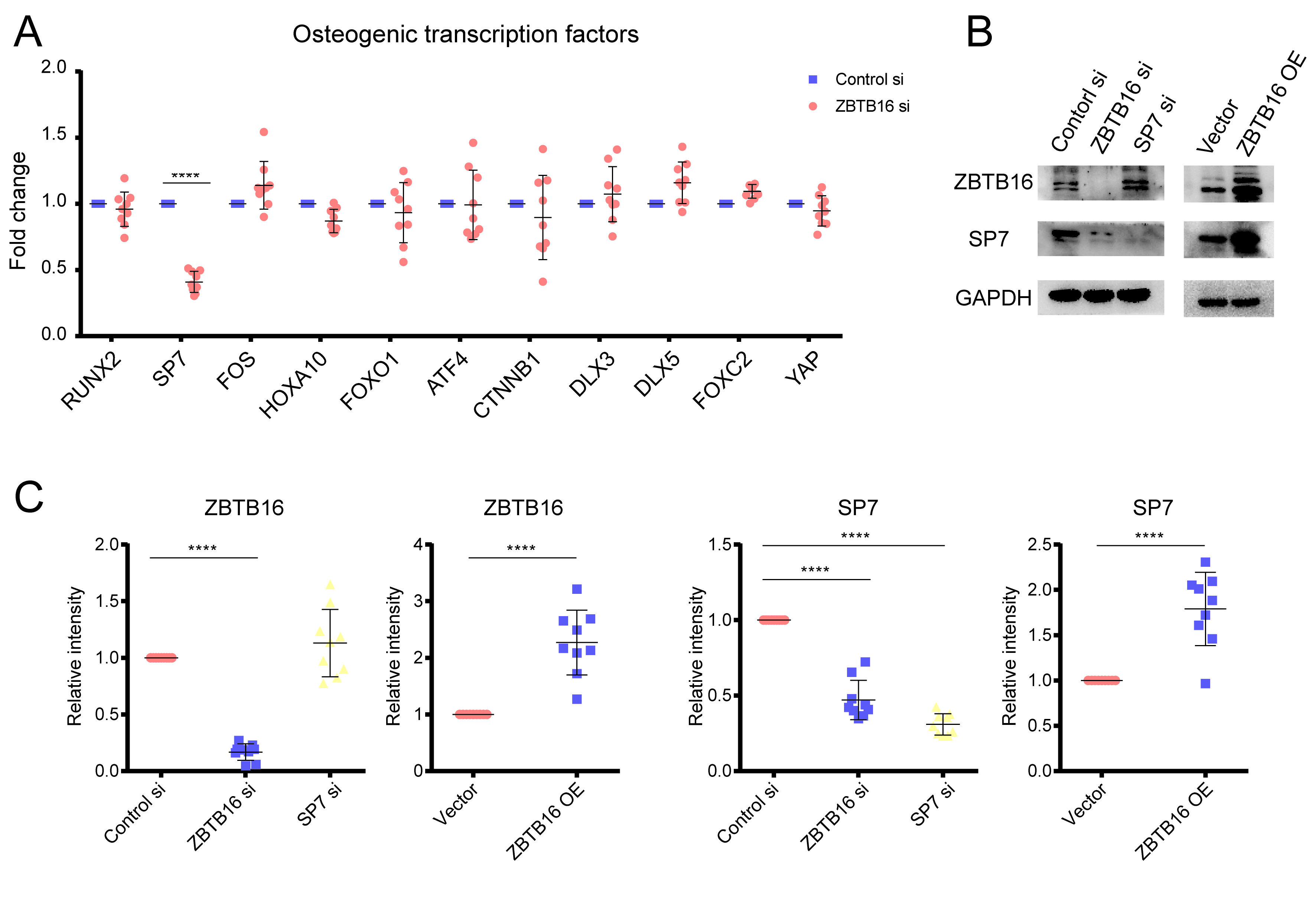

Supplement: Supplementary file 6 — Supplementary Figure 4 [file 41413_2023_267_MOESM6_ESM.tif]
