# Supplementary material for: Super enhancers targeting ZBTB16 in osteogenesis protect against osteoporosis
Source: Bone Res. 2023 Jun 7;11:30. doi: 10.1038/s41413-023-00267-8 (PMC10244438; doi:10.1038/s41413-023-00267-8)
Supplement: Supplementary file 8 — Supplementary Figure 6 [file 41413_2023_267_MOESM8_ESM.docx]

Supplementary Table 1 Clinical features of control and osteoporosis subjects

| Clinical features | Group | | P value |
| --- | --- | --- | --- |
|  | Control | Osteoporosis |  |
| All subjects (n, %) | 21 (100) | 17 (100) |  |
| Gender |  |  |  |
| Male | 12 | 9 | 0.7956 |
| Female | 9 | 8 |  |
| Age (mean ± sd) | 30.71±3.52 | 73.17±4.03 | <0.0001 |
| T score | -0.41±0.13 | -2.61±0.33 | <0.0001 |
| BMI (kg/m2) | 20.70±2.10 | 21.20±1.79 | 0.4479 |
