# Supplementary material for: Super enhancers targeting ZBTB16 in osteogenesis protect against osteoporosis
Source: Bone Res. 2023 Jun 7;11:30. doi: 10.1038/s41413-023-00267-8 (PMC10244438; doi:10.1038/s41413-023-00267-8)
Supplement: Supplementary file 13 — Supplementary Table 5 [file 41413_2023_267_MOESM13_ESM.docx]

**Supplementary Fig. 1 Identification of SEs in hBMMSCs, immortal TERT4-MSCs and hFOB 1.19 cells**

Enhancers are ranked by the degree of ChIP-seq signal in each dataset. Points higher than the point where the slope is greater than 1 are defined as SEs.

**Supplementary Fig. 2 Verification of the efficacy of siRNAs and overexpression plasmids**

**(A)** Efficacy of BRD4 siRNAs on the relative mRNA expression of BRD4.

**(B)** Efficacy of the BRD4 overexpression plasmid on the relative mRNA expression of BRD4.

**(C)** Immunoblot analysis verifying the knockdown and overexpression efficacy of BRD4 siRNA and overexpression plasmid. Scatter plots showing the relative abundance of BRD4.

**(D)** Efficacy of ZBTB16 siRNA knockdown.

**(E)** Efficacy of the ZBTB16 overexpression plasmid.

**(F)** Immunoblot analysis verifying the knockdown and overexpression efficacy of ZBTB16 siRNA and overexpression plasmid. Scatter plots showing the relative abundance of ZBTB16.

**(G)** Efficacy of RPAP2 siRNAs on the relative mRNA expression of RPAP2.

**(H)** Efficacy of the RPAP2 overexpression plasmid on the relative mRNA expression of RPAP2.

**(I)** Immunoblot analysis verifying the knockdown and overexpression efficacy of RPAP2 siRNA and overexpression plasmid. Scatter plots showing the relative abundance of RPAP2.

**(J)** Efficacy of SP7 siRNAs on the relative mRNA expression of SP7.

**(K)** Immunoblot analysis verifying the knockdown efficacy of SP7 siRNA. Scatter plot showing the relative abundance of SP7.

The statistical data are represented as the means ± SEMs, n = 9, *P < 0.05, **P < 0.01, ***P < 0.005, ****P < 0.001.

**Supplementary Fig. 3 Verification of the effect of JQ1 on MSC proliferation and osteogenic differentiation**

**(A)** Scatter plot of CCK-8 analysis data showing the effect of JQ1 on MSC proliferation.

**(B)** ARS staining and quantification of MSCs treated with DMSO or JQ1. The upper row is the microscopic field, and the lower row is the general field. Scatter plot showing ARS quantification.

**(C)** ALP staining and activity levels in MSCs treated with DMSO or JQ1. The upper row is the microscopic field, and the lower row is the general field. Scatter plot showing ALP activity.

The statistical data are represented as the means ± SEMs, n = 9, *P < 0.05, **P < 0.01, ***P < 0.005, ****P < 0.001.

**Supplementary Fig. 4 Construction strategy and altered bone repair capacity of Brd4 CKO mice**

**(A)** CKO strategy for reducing bone-specific Brd4 expression.

**(B)** Scatter plots showing the calvarial and femoral defects in the *Brd4*^fl/fl^ and *Brd4*^fl/fl^ *Prx1*-cre mice.

The statistical data are represented as the means ± SEMs, n = 5, *P < 0.05, **P < 0.01, ***P < 0.005, ****P < 0.001.

**Supplementary Fig. 5 ZBTB16 regulated the expression of the early osteogenic TF SP7**

**(A)** ZBTB16 knockdown regulates the expression of osteogenic TFs.

**(B)** Immunoblot analysis showing that ZBTB16 acts upstream of SP7. ZBTB16 knockdown and overexpression downregulate and upregulate SP7, respectively, but SP7 knockdown shows no effect on ZBTB16 expression.

**(C)** Scatter plots showing the expression of ZBTB16 and SP7 in immunoblot analysis.

The statistical data are represented as the means ± SEMs, n = 9, *P < 0.05, **P < 0.01, ***P < 0.005, ****P < 0.001.

**Supplementary Fig. 6 Collateral decrease in Zbtb16 expression and therapeutic effect of Zbtb16 on disrupted bone repair**

**(A)** Immunofluorescence showing Brd4 and Zbtb16 expression in femurs of the *Brd4*^fl/fl^ and *Brd4*^fl/fl^ *Prx1*-cre mice.

**(B)** Scatter plots showing the calvarial and femoral defects of the *Brd4*^fl/fl^ *Prx1*-cre mice injected with rAAV9 vector or bone-targeting Zbtb16-overexpressing rAAV9.

The statistical data are represented as the means ± SEMs, n = 5, *P < 0.05, **P < 0.01, ***P < 0.005, ****P < 0.001.
